# The interactions between integrin α_5_β_1_ of liver cancer cells and fibronectin of fibroblasts promote tumor growth and angiogenesis

**DOI:** 10.7150/ijbs.72367

**Published:** 2022-08-01

**Authors:** Zheng Peng, Meng Hao, Haibo Tong, Hongmei Yang, Bin Huang, Zhigang Zhang, Kathy Qian Luo

**Affiliations:** 1 Faculty of Health Sciences, University of Macau, Taipa, Macao SAR, China.; 2 State Key Laboratory of Oncogenes and Related Genes, Shanghai Cancer Institute, Renji Hospital, School of Medicine, Shanghai Jiao Tong University, Shanghai, China.; 3 Ministry of Education-Frontiers Science Center for Precision Oncology, University of Macau, Taipa, Macao SAR, China.

**Keywords:** Hepatocellular carcinoma, tumor growth, fibronectin, integrin-α_5_, integrin-β_1_, angiogenesis

## Abstract

Hepatocellular carcinoma (HCC) progression is closely related to pathological fibrosis, which involves heterotypic intercellular interactions (HIIs) between liver cancer cells and fibroblasts. Here, we studied them in a direct coculture model, and identified fibronectin from fibroblasts and integrin-α_5_β_1_ from liver cancer cells as the primary responsible molecules utilizing CRISPR/Cas9 gene-editing technology. Coculture led to the formation of 3D multilayer microstructures, and obvious fibronectin remodeling was caused by upregulated integrin-α_5_β_1_, which greatly promoted cell growth in 3D microstructures. Integrin-α_5_ was more sensitive and specific than integrin-β_1_ in this process. Subsequent mechanistic exploration revealed the activation of integrin-Src-FAK, AKT and ERK signaling pathways. Importantly, the growth-promoting effect of HIIs was verified in a xenograft tumor model, in which more blood vessels were observed in bigger tumors derived from the coculture group than that derived from monocultured groups. Hence, we conducted triculture by introducing human umbilical vein endothelial cells, which aligned to and differentiated along multilayer microstructures in an integrin-α_5_β_1_ dependent manner. Furthermore, fibronectin, integrin-α_5_, and integrin-β_1_ were upregulated in 52 HCC tumors, and fibronectin was related to microvascular invasion. Our findings identify fibronectin, integrin-α_5_, and integrin-β_1_ as tumor microenvironment-related targets and provide a basis for combination targeted therapeutic strategies for future HCC treatment.

## Introduction

HCC comprises 75%-85% of primary liver cancer cases and is the leading cause of cancer-related mortality 1-3. The development of HCC is believed to be closely related to pathological fibrosis 4, 5, which involves extracellular matrix (ECM) remodeling and heterotypic intercellular interactions (HIIs) between liver cancer cells and surrounding fibroblasts 6, 7. Currently, many studies have focused on this field in the hope of finding key tumor microenvironment (TME)-related targets for combination targeted therapy 6-13, given the unsatisfactory outcomes of current therapies.

As a major product of fibroblasts and a versatile ECM component in the TME, fibronectin (Fn) carries out many pivotal physiological and pathological functions, since it provides a provisional scaffold for the assembly of other ECM proteins 12, 14-20. Integrin-α_5_β_1_, as the primary fibronectin receptor and a widely distributed dimeric transmembrane protein 21, can serve as a cellular “hand” that exerts tensional force upon naturally coiled fibronectin to expose cryptic binding domains inside fibronectin 21-28, which could greatly promote fibrillogenesis 14, 29. Importantly, vascular endothelial growth factor-A (VEGFA) can bind to these exposed domains 30-35, and exert a more potent proangiogenic effect than free VEGFA 20, 36, 37. Taken together, the above findings suggest the neglected roles of HIIs between fibronectin from fibroblasts and integrins from cancer cells in the development of pathological fibrosis and angiogenesis in HCC.

To investigate the HIIs between liver cancer cells and fibroblasts, we established a direct coculture system. After finding that HIIs caused 3D multilayer microstructure formation, we performed CRISPR/Cas9 gene-editing to determine primary responsible targets and mechanistically analyzed how these targets exert their effects. *In vivo* experiments were performed to confirm our findings. Then, focusing on the biological functions of HIIs, we introduced human umbilical vein endothelial cells (HUVECs) and further explored the roles of these targets. Finally, clinical significance was explored by immunohistochemistry staining (IHC-staining) and KMplot database analysis.

## Material and methods

### Cell lines and cell culture

MEFs and MEF-FN^-/-^ cells were derived from mouse kidneys and immortalized by lentiviral transduction of SV40 (Simian vacuolating virus 40) large T antigen, and generously provided by Professor Reinhard Fässler and his assistant Dr. Ralph Böttcher (Max-Planck Institute for Biochemistry, Martinsried, Germany). The human hepatocellular carcinoma cell line HepG2 and the HUVEC cell line CRL-1730™ were purchased from ATCC (American Type Culture Collection; Rockville, MD, USA). Huh7 cells were kindly provided by Prof. Chuxia Deng from University of Macau. HepG2-α_5_^-/-^, HepG2-β_1_^-/-^, HUVEC-α_5_^-/-^, and HUVEC-β_1_^-/-^ were single cell-derived gene-knockout clones generated by the CRISPR/Cas9 system. All monocultured and cocultured cells were cultured in DMEM (Dulbecco's modified Eagle's medium) containing 10% fetal bovine serum (FBS) and 100 U/ml penicillin-streptomycin (Gibco; Thermo Fisher Scientific, Inc., Waltham, MA, USA) and maintained in a humidified incubator with 5% CO_2_ at 37 °C.

### Reagents

Mowiol® 4-88 powder was ordered from Sigma-Aldrich (St. Louis, MO, USA). Sunitinib, apatinib, dasatinib, dactolisib, trametinib, and Y15 were purchased from Selleck Chemicals (Houston, TX, USA). Recombinant human VEGFA (rhVEGFA) was ordered from R&D Systems (Minneapolis, MN, USA).

### Coculture and triculture systems

After trypsinization and cell counting, liver cancer cells and MEF cells were mixed at a ratio of 1:1 for the coculture assay. For triculture, liver cancer cells, MEF cells, and HUVCs were mixed at a ratio of 5:3:2 before being seeded in cell culture dishes.

### Cell density assay

Cells were plated at 2.5 × 10^5^ cells/dish (60 mm in diameter) and cultured for 1 to 10 days in DMEM supplemented with 10% FBS. The culture medium was changed every day, and cell numbers were counted. Cell density was calculated by dividing the total cell number by the total culture surface area. Each value represents the mean ± S.D. of triplicate experiments.

### Protein extraction and Western blot

After washing cells with PBS, cellular proteins were extracted using RIPA lysis buffer supplemented with protease and phosphatase inhibitors (Sigma-Aldrich). After sonication and centrifugation, the supernatant was collected and quantified using Protein Assay Dye Reagent concentrate (Bio-Rad). After denaturation (95 °C, 5 min), equal amounts of protein (30 μg) from different samples were separated by SDS-PAGE and then electrotransferred (Mini-PROTEAN system, Bio-Rad) to a nitrocellulose membrane (Bio-Rad). After blocking with 5% Blotting-Grade Blocker (Bio-Rad) for 1 hr, the membrane was probed with a specific primary antibody (4°C, overnight) and then incubated with the HRP-conjugated secondary antibody (room temperature, 1 hr). Finally, after incubation with chemiluminescent substrate (Clarity Western ECL Substrate, Bio-Rad), the membrane was exposed using a ChemiDoc™ Touch Imaging System (Bio-Rad). The primary antibodies used in this study are listed in Table S2.

### Fluorescence live-cell microscopy

Fluorescence live-cell imaging was performed using a Zeiss Axio Observer microscope equipped with a Zeiss Axiocam 512 monocamera and a set of objectives and filters, which could capture the right size pictures and separate signals from different channels, and captured images were then analyzed and exported by ZEN imaging software (Carl Zeiss Vision GmbH, München, Germany).

### Two-photon fluorescence microscopy

Two-photon fluorescence imaging was performed using a Nikon A1RMP confocal and multiphoton microscope equipped with lasers (820-1300 nm), NDD detectors, and a set of objectives and then analyzed and exported by NIS-Elements AR 5.2 software (Nikon Instrument Inc., Melville, NY).

### Immunofluorescence staining (IF-staining)

Cells were washed in PBS before fixation with 4% paraformaldehyde for 15 min and then permeabilized with 0.2% Triton-X 100 for 10 min at room temperature (RT). After blocking with 3% BSA for 1 hr, cells were incubated with primary antibody at the recommended dilution, followed by Alexa-Fluor 594- or 488-conjugated secondary antibodies (Invitrogen, Thermo Fisher Scientific) at a 1:100 dilution for 1 hr. Nuclei were labeled with Hoechst-33342 (Thermo Fisher Scientific) before cells were mounted onto a microscope slide using Mowiol® 4-88. Finally, IF-staining images were captured with a Zeiss Axio Observer microscope. The primary antibodies used in this study are listed in Table S2.

### Enzyme-linked immunosorbent assay (ELISA)

The protein levels of mouse VEGFA in the cell culture supernatants were quantified using an ELISA kit from R&D Systems (Minneapolis) according to the manufacturers' instructions. Briefly, after capture antibody coating overnight and subsequent blocking for 1 hr, the prepared 96-well microplates were added to 100 μl of sample or standards and incubated for 2 hr at RT. After 3 washes, the detection antibodies were added to each well and incubated for 2 hr at RT. After 3 washes, streptavidin-HRP was added to each well and incubated for 20 min at RT. After 3 washes, substrate solution was added and incubated for 20 min at RT. Finally, the stop solution was added to each well. After gentle mixing, the optical density value of each well was determined by a plate reader immediately at 450 nm and 540 nm.

### Wound healing plus IF-staining

Liver cancer cells and MEF-clover cells were seeded separately (3000 cells per well) in a 2 well silicone culture-insert (iBidi, Martin Reid, Gräfelfing, Germany) mounted on a glass slide to create a 500 μm cell-free gap between these two cell types. The culture-insert was then removed on day 3 after they reached confluence, and the glass slide was placed in a culture dish to allow these two cell types to grow toward each other. Finally, the glass slide was removed from the dish on day 5 and subjected to IF-staining procedures.

### Species-specific primers

First, the human and mouse mRNA sequences of genes of interest were obtained from the NIH website. The alignment of human and mouse mRNA sequences was conducted by using SnapGene software (Version 3.1; GSL Biotech, snapgene.com), with differentiated parts marked out. At least 3 independent species-specific primers were designed on the basis of differentiated mRNA sequences if possible. The specificity of the primers was first examined using the “primer designing tool” to check whether there were products in the other specie's genome. Then, these primer sequences were sent to and synthesized by Beijing Genomics Institute (BGI, China) in Guangzhou. Finally, the validation of these primers was performed by checking the melt curves after RT-qPCR and running electrophoresis in 1.5% agarose gels with the q-PCR products. The primers used in this study are listed in Table S3.

### Real-time quantitative polymerase chain reaction (RT-qPCR)

First, total RNA was extracted using Trizol^®^ (Thermo Fisher Scientific) and isopropanol (Sigma-Aldrich) precipitation and then reverse transcribed into cDNA by an iScript™ cDNA Synthesis Kit (Bio-Rad). Finally, RT-qPCR was conducted in triplicate using cDNA, primers, and iTaq™ Universal SYBR^®^ Green Supermix (Bio-Rad) on a CFX96 Touch™ Real-Time PCR Detection System (Bio-Rad). Fold changes in mRNA levels of genes of interest were compared using the ΔΔC_t_ method with the ΔΔC_t_ of GAPDH as the internal control.

### Human hepatocellular carcinoma xenograft tumor model in nude mice

All animal experiments were conducted in accordance with the guidelines approved by the Animal Research Ethics Committee of the University of Macau. Five-week-old nude mice were injected subcutaneously with 2 million cells in the right flank. Body weight and xenograft volume were monitored every 3 days beginning on day 5. Xenograft volume (mm^3^) was calculated by using the following formula: π/6 × length × width^2^. All mice were sacrificed on day 29, and tumor xenografts were dissected using a surgical blade and weighed before taking images by a digital camera and an Olympus MVX10 macro-zoom fluorescence microscope (Olympus, Hamburg, Germany). Finally, xenografts were fixed in 4% paraformaldehyde and embedded in paraffin blocks before tissue sectioning.

### Immunohistochemistry staining (IHC-staining)

Tissue microarray (TMA) slides were generously provided by Prof. Zhang Zhigang (Shanghai Cancer Institute, China). After deparaffinization and hydration in Milli-Q water, antigens were retrieved by boiling TMA slides in citrate buffer for 20 min. Then, endogenous peroxidase activity and nonspecific binding activity were blocked before incubation with the primary antibody at the recommended dilution (4 °C, overnight) in a wet box. Finally, immunoreactivity was visualized using the Mouse and Rabbit specific HRP/DAB (ABC) Detection IHC Kit (Abcam, Cambridge, MA, USA) in accordance with the manufacturer's instructions. Nuclei were counterstained with hematoxylin (Sigma-Aldrich). Images were taken by a NanoZoomer S60 slide scanner (Hamamatsu Photonics, Shizuoka, Japan) at a magnification of 20×. The expression levels of corresponding targets were determined by quantitative analysis. IHC scores were graded according to the percentage of stained cells as previously described 38. Briefly, the scoring was assigned as follows: the percentage scores: 0, <5% of positively stained cells; 1, 5-50%; 2, 51-100%; the intensity scores: 0, absent or faint; 1, weak; 2, moderate; 3, strong.

### Tube formation assay (TFA)

A total of 2.5 × 10^5^ HUVEC-i670 cells were resuspended in 50 μl medium containing 0.5% FBS and plated onto Matrigel-coated µ-slides (iBidi). After incubation for 6 hr, images were taken on a Zeiss Axio Observer microscope.

### Image quantitative analysis

For quantitative analysis of the percentage of area occupied by MEF-clover cells, images were analyzed by ImageJ 1.53c software (National Institute of Health, Bethesda, MD, USA) to obtain the percentage of area of interest. For quantitative analysis of the colocalized area of two types of fluorescent cells, images were analyzed by AutoQuant X3 software (Media Cybernetics Inc., Rockville, MD, USA) to obtain the percentage of colocalized area.

### Quantitative analysis of angiogenesis characteristics

Far red channel live-cell fluorescence images were analyzed by AngioTool software (National Cancer Institute, Bethesda, MD, USA). From the analysis, the number of junctions, number of tubules, total tubule length, and average tubule length are presented.

### Lentivirus production and transfection

Core plasmids were transfected into Lenti-X 293T cells by polyethylenimine (PEI, Polysciences, Warrington, PA, USA) to produce lentivirus, together with envelope plasmid (dR8.2) and virus packaging plasmid (VSV-G). Then, the produced viral particles were concentrated and collected at 48 hr and 72 hr and added to target cells for transfection. The primary core plasmids used in this study are listed in Table S4.

### Fluorescence activated cell sorting (FACS)

The fluorescent protein-transfected cell suspension was kept at 1-10 million/ml in PBS after trypsinization. Cells were filtered immediately before sorting by a BD FACSAria™ III digital flow cytometer (San Diego, CA, USA). During the sorting process, the top 10% of cells were collected for fluorescence intensity. Then, this process was repeated after the collected cells reached enough numbers for the next round of sorting. On the whole, 2-4 rounds of sorting yielded a fluorescent cell line.

### Generation of single cell-derived gene-knockout clones

After trypsinization, transfected cells were counted and diluted to a concentration of 200 cells/ml, and serial dilutions were performed in a flat bottom 96-well plate. Every well was observed under a microscope, and wells containing a single cell were marked. Allow single cells to expand to microcolonies. Split cells when they reach confluence. Continue expanding them until they proliferate to a sufficient number. Finally, the gene-knockout effect was validated using Western blotting.

### Statistical analysis

All experiments were conducted at least 3 times. All the data are presented as the mean ± SD (standard deviation). The difference between groups was analyzed using the relevant test through GraphPad Prism 8 (GraphPad Software Inc., San Diego, CA, USA). The following probability values were deemed statistically significant: *P<0.05, **P<0.01, ***P<0.001, ****P<0.0001.

## Results

### Coculture of liver cancer cells and MEFs led to the formation of 3D multilayer microstructures

Inspired by our previous study 39, to determine the HIIs between liver cancer cells and fibroblasts, we established a coculture system using liver cancer cells and mouse embryo fibroblasts (MEFs). To distinguish these two cell types, we separately introduced plasmids to express tdTomato (tdT) and clover and obtained HepG2-tdT, Huh7-tdT, and MEF-clover cell lines. Then, we observed the HIIs between two cell types by comparing coculture and monoculture systems using live-cell fluorescence images of the same spot over 10 sequential days. A ratio of 1:1 between the two cell types was set as the best combination, and coculture without fluorescent protein expression was performed to exclude possible confounding factors (Fig. S1A-D, and Fig. S3A).

**Figure 1:**
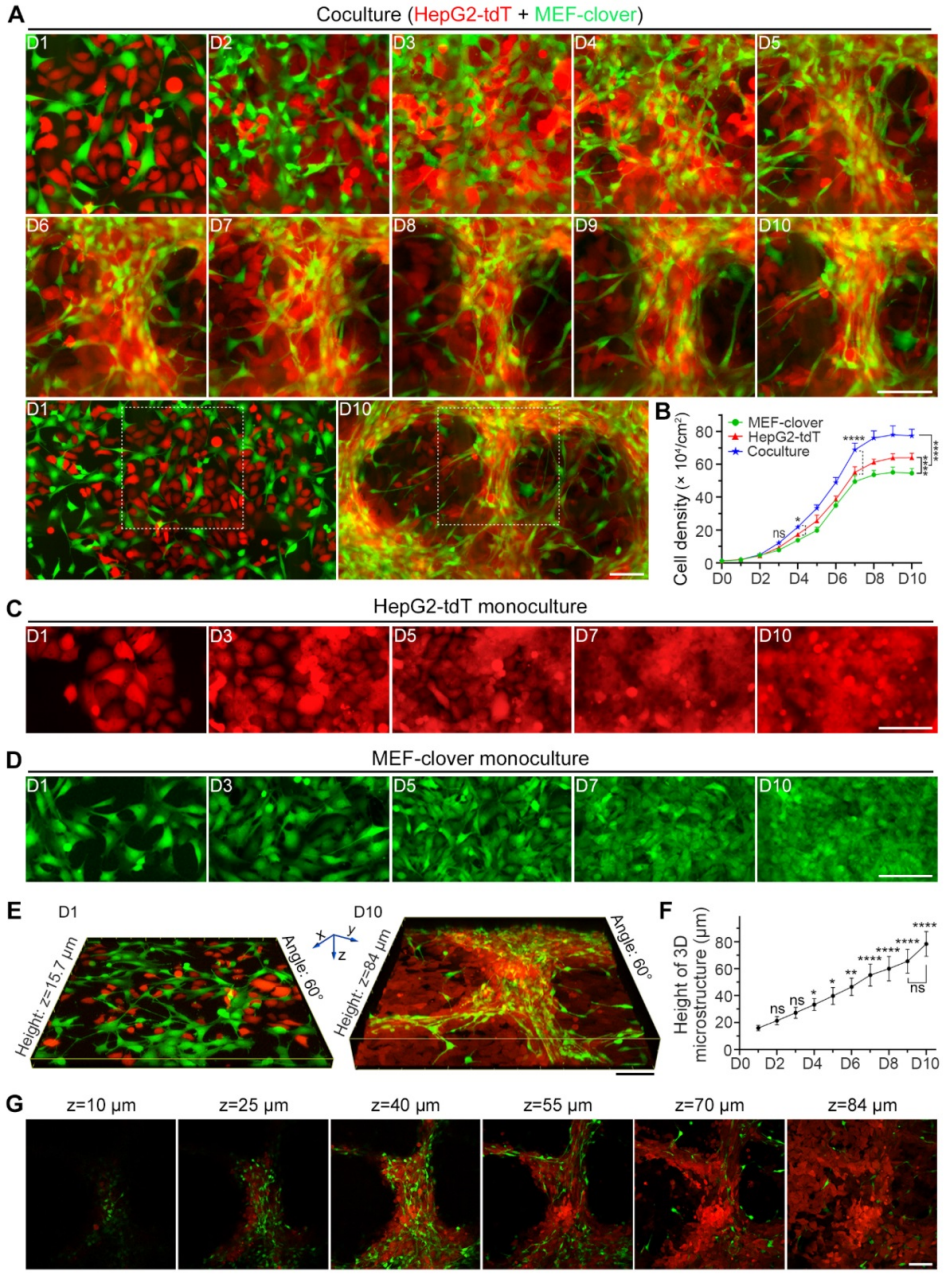
Coculture of HepG2 cells and MEFs led to the formation of 3D multilayer microstructures. (**A**) Representative live-cell fluorescence images demonstrating the formation of 3D multilayer microstructures from day 1 to day 10. HepG2-tdT and MEF-clover cells were cocultured at a ratio of 1:1. Gridded glass coverslips were employed to take images at the same spots. Zoomed-out views of the images on day 1 and day 10 are shown below. (**B**) A cell density curve of monoculture and coculture systems from day 1 to day 10. HepG2-tdT and MEF-clover cells were monocultured or cocultured in 2.5 × 10^5^ cells/60-mm culture dishes, and the cell density was assessed every day. Two-way ANOVA and Tukey's multiple comparison test were performed. (**C** and **D**) Representative images of monocultured HepG2-tdT (**C**) and MEF-clover cells (**D**) at the same spots. (**E**) Representative 3D XYZ reconstructed images of cocultured cells on day 1 and day 10. Images were generated from Z-stack images taken on a two-photon microscope. (**F**) A height curve of 3D multilayer microstructures formed during coculture. Height data were extracted from the 3D volume-rendering images. One-way ANOVA and Tukey's multiple comparison test were performed, using the height on day 1 as the reference point. (**G**) XY-plane images at different heights of a 3D multilayer microstructure formed in coculture on day 10. Scale bars, 100 µm. All data are the mean ± SD from 3 independent experiments. * *P*<0.05, ** *P*<0.01, **** *P*<0.0001, and ns, not significant.

From the chronological images in Fig. 1A, we observed that after the introduction of HIIs by coculture, 3D multilayer microstructures began to form on day 4 and became obvious after day 5. The overall zoomed-out images corresponding to the enlarged parts on day 1 and day 10 clearly reveal the dramatic effects induced by HIIs. However, monocultured cells formed only small aggregates (Fig. 1C and D). Moreover, from the cell density comparison shown in Fig. 1B, we observed that coculture slightly increased cell density on day 4 and had a more significant effect after day 7. On day 10, the cocultured cell density was increased by more than 20% compared with monocultured cells, probably because 3D microstructures improved growth environment.

To characterize microstructures, we reconstructed 3D volume-rendering images and found that in contrast to their tendency to spread in monoculture, the cocultured MEFs tended to gather, and HepG2 cells displayed higher cell density surrounding MEFs, which caused two cell types to converge. Interestingly, unlike the wide distribution of HepG2 cells, MEFs were mainly distributed along 3D multilayer microstructures after day 5, suggesting their active role in microstructure formation. Moreover, the morphology of most fibroblasts shifted from a flat spindle-like shape to an elongated shape (Fig. 1A, Fig. S2A and E).

Next, we generated a height curve using the height extracted from two-photon images (Fig. 1E and F), and observed that compared with the height on day 1 (15.9 μm), the height had doubled around day 4 (33.3 μm) and peaked on day 10 (78.4 μm), which reflected the effects of HIIs as shown in Fig. 1A. However, the height fluctuated around 35.9 μm and 20.9 μm on day 10 for monocultured HepG2-tdT and MEF-clover cells, respectively (Fig. S2B-D).

To determine the topological relationship between two cell types inside 3D microstructures, XY-plane images at different depths on day 10 were examined and are shown in Fig. 1G. Combined with results in Fig. S2A, we observed that the relationship between the two cell types shifted from their random distribution to their interweaving in 3D microstructures, with MEFs mainly distributed in upper layers, while HepG2 cells mainly distributed in bottom layers.

Similar results were obtained in the coculture of Huh7 cells and MEFs (Fig. S3A-D). Taken together, we concluded that HIIs promoted the aggregation of liver cancer cells and fibroblasts during coculture, and caused the formation of 3D multilayer microstructures.

### Within 3D microstructures, integrin-α_5_ and integrin-β_1_ were upregulated in liver cancer cells, while fibronectin and collagen I assembly were increased in fibroblasts

Integrins and ECM proteins are probably involved in microstructure formation, since they are the primary players in the formation of focal adhesions which bring different cells together to form microstructures. Then, we performed Western blot (WB) assays to determine the expression of major integrins and ECM proteins.

**Figure 2:**
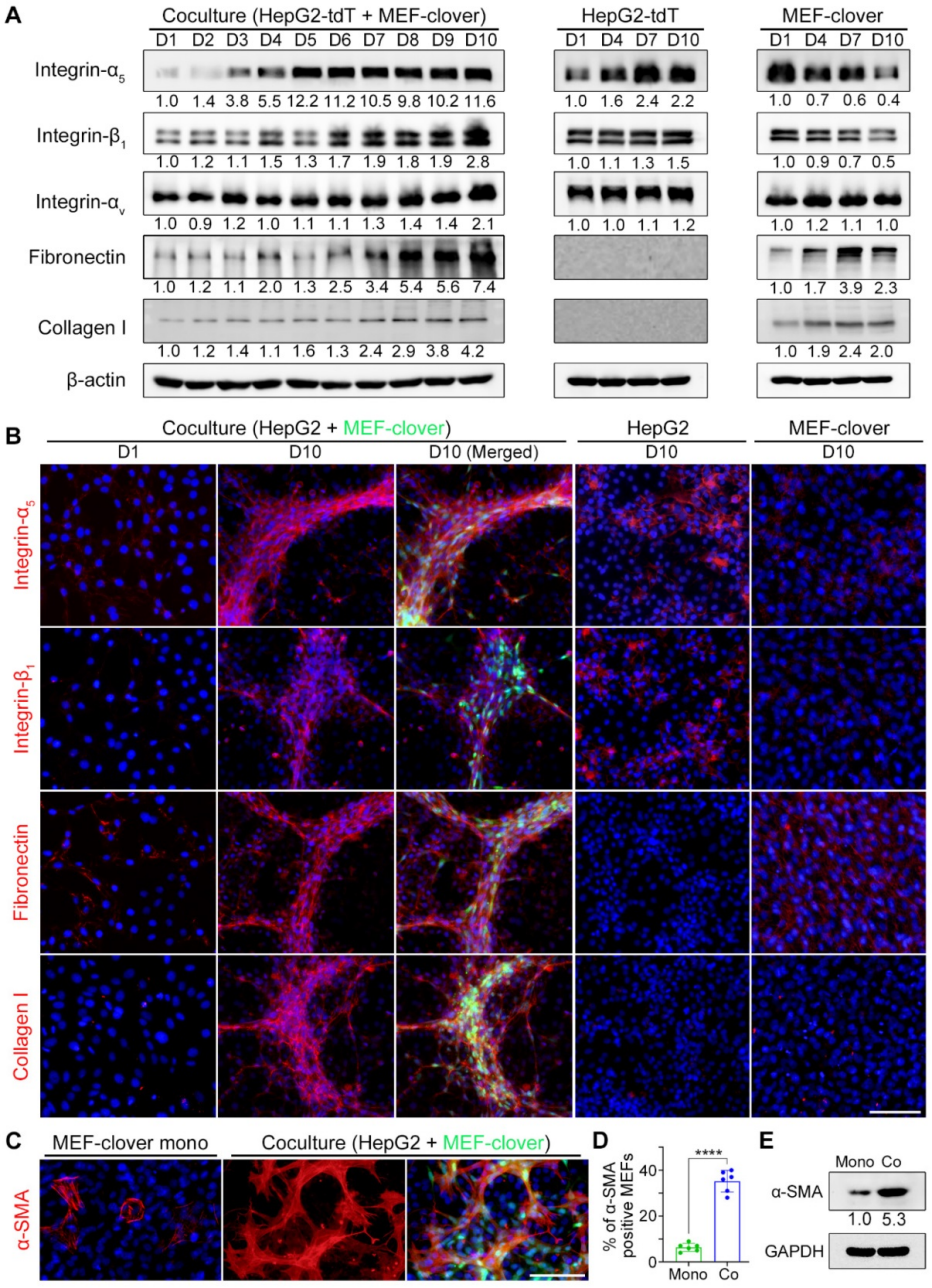
Integrins and ECM proteins were enriched in 3D multilayer microstructures with more activated fibroblasts. (**A**) WB results showing the levels of related proteins in cocultured cells and monocultured cells from day 1 to day 10. (**B**) Representative IF-staining images of related proteins in cocultured cells and monocultured cells. (**C** and **D**) Representative IF-staining images of α-SMA in monocultured MEF-clover cells and coculture cells on day 5 (**C**), and subsequent quantification of the proportion of α-SMA-positive MEFs under two conditions (**D**). The results were quantified based on the same area in the 20 × observation field (2120 × 1416 pixels, 0.31 µm × 0.31 µm per pixel) from 3 independent experiments, and data are shown as the mean ± SD. Unpaired t test was performed. **** *P*<0.0001. (**E**) WB results of the levels of α-SMA in monocultured MEF-clover cells and cocultured cells (HepG2 and MEF-clover cells) on day 10. Nuclei were visualized using Hoechst-33342 staining. Scale bars, 100 µm.

From WB results in Fig. 2A, we saw integrin-α_5_ displayed a higher increase compared with integrin-β_1_ or integrin-α_V_ during coculture, in which integrin-α_5_ increased by 11.6-fold, while integrin-β_1_ and integrin-α_V_ increased by 2.8- and 2.1-fold on day 10, respectively; fibronectin showed a higher increase than collagen I on day 10 (7.4-fold vs. 4.2-fold). Moreover, integrin-α_5_ upregulation occurred earlier than other integrins and quickly reached its maximum level of 12.2-fold on day 5. Similarly, fibronectin upregulation occurred earlier than collagen I upregulation. These results indicated that integrin-α_5_ and fibronectin are probably the primary mediators.

Based on WB results in Fig. 2A, we knew fibronectin and collagen I were mainly from fibroblasts, as supported by q-PCR results (Fig. S4A) using species-specific primers that only recognize either human- or mouse-derived corresponding mRNAs. For integrin-α_5_ and integrin-β_1_, although both types of cells displayed a basal protein level, mRNA levels of both genes from MEFs in coculture displayed downward trends as in monoculture. However, mRNA levels of both genes in cocultured HepG2 cells showed upward trends, with ITGA5 and ITGB1 mRNA levels on day 10 increasing by 469.4% and 42.9% compared with day 1, respectively. Moreover, both genes' mRNA levels in cocultured HepG2 cells displayed significant increases compared with monocultured HepG2 cells on day 10, as ITGA5 displayed a higher increase than ITGB1 (129.4% vs. 8.8%) (Fig. S4A). These results illustrated that upregulated integrin-α_5_ and integrin-β_1_ were mainly from cocultured HepG2 cells, and integrin-α_5_ was more sensitive to HIIs than integrin-β_1_.

Since our interest is in microstructure formation, the structural and distributional changes of these proteins during microstructure formation at different timepoints were investigated by immunofluorescence staining (IF-staining). Monocultured HepG2 and MEF-clover cells on day 10 were adopted as controls (Fig. S4B and Fig. 2B). Little bleed-through was observed using Alexa-Fluor 594 labeled secondary antibodies and suitable filter sets (Fig. S5A). From IF results, we observed that integrin-α_5_ and integrin-β_1_ underwent dramatic distributional changes during coculture, shifting from a scattered pattern to a clustered pattern with enriched signals distributed in 3D microstructures, while in monoculture, the weak signals were irregularly distributed with small aggregates. In addition to similar distributional changes, fibronectin and collagen I underwent dramatic structural changes, shifting from small thin fibers to long thick bundles, while no such changes were observed in monocultures (Fig. 2B and Fig. S5B). Similar results were also obtained in the IF staining and WB results of Huh7 and MEFs coculture (Fig. S6 and Fig. S7). These changes clearly illustrated the existence of HIIs and its effects on fibronectin and collagen I remodeling. The results were repeated using cells not expressing fluorescent proteins (Fig. S5B and Fig. S7B).

To highlight fibroblasts, we merged the MEF-clover cells with target proteins. Interestingly, we observed good colocalization (Fig. 2B and Fig. S6), suggesting the active role of fibroblasts in microstructure formation. Additionally, the distribution pattern of fibroblasts in coculture was also confirmed by IF-staining using cells not expressing fluorescent proteins through their elliptical, speckled nuclei (Fig. S5B-D).

Furthermore, results from wound healing plus IF-staining experiments showed that where the two cell types converged, integrin-α_5_, integrin-β_1_, and fibronectin signals were obviously stronger than those of either cell type's territory (Fig. S8A-C). These results further verified that HIIs are the driving force for these proteins' upregulation.

Since MEFs underwent significant morphological changes during coculture, we also tested related markers and found that fibroblast growth factor receptor-1 (FGFR1) and α-smooth muscle actin (α-SMA) were upregulated in cocultured MEFs compared with monocultured MEFs. Importantly, from the quantified results of α-SMA, we found that the percentage of α-SMA-positive MEFs was significantly increased from 6% in monoculture to 35% in coculture, which was supported by a 5.3-fold protein level increase (Fig. 2C-E and Fig. S8D).

Collectively, above results suggest that fibronectin from fibroblasts and integrin-α_5_ and integrin-β_1_ from liver cancer cells are probably the main players in 3D microstructure formation.

### Fibronectin, integrin-α_5_ and integrin-β_1_ are essential in promoting 3D microstructure formation

**Figure 3:**
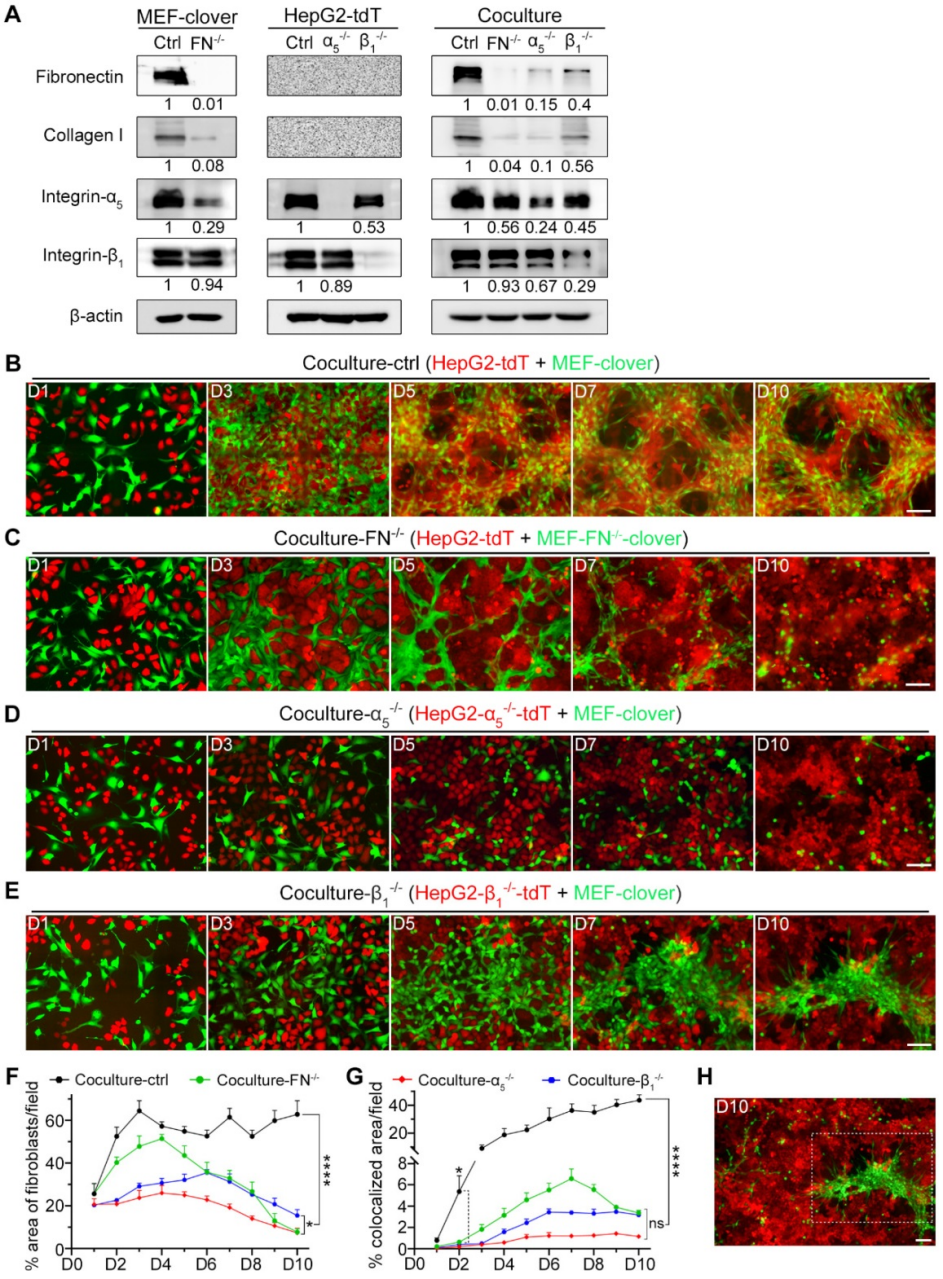
The formation of 3D multilayer microstructures required fibronectin, integrin-α_5_, and integrin-β_1_. (**A**) WB analysis of the levels of fibronectin, collagen I, integrin-α_5_, and integrin-β_1_ in the following samples: MEF-clover cells and MEF-FN^-/-^-clover cells; HepG2-tdT cells, HepG2-α_5_^-/-^-tdT cells, and HepG2-β_1_^-/-^-tdT cells; cocultured HepG2-tdT and MEF-clover cells (coculture-ctrl), cocultured HepG2-tdT and MEF-FN^-/-^-clover cells (coculture-FN^-/-^), cocultured HepG2-α_5_^-/-^-tdT and MEF-clover cells (coculture-α_5_^-/-^), and cocultured HepG2-β_1_^-/-^-tdT cells and MEF-clover cells (coculture-β_1_^-/-^). (**B** to **E**) Representative live-cell fluorescence images of coculture-ctrl (**B**), coculture-FN^-/-^ (**C**), coculture-α_5_^-/-^ (**D**), and coculture-β_1_^-/-^ (**E**) groups at the same spots from day 1 to day 10. (**F**) The percentages of XY-plane growth space occupied by fibroblasts per 10 × observation field (4248 × 2832 pixels, 0.31 µm × 0.31 µm per pixel) were measured using ImageJ in the following four coculture groups: coculture-ctrl, coculture-FN^-/-^, coculture-α_5_^-/-^, and coculture-β_1_^-/-^. (**G**) The percentages of colocalized area between two cell types per 10 × observation field were measured in the four coculture groups with AutoQuant X3 software. (**H**) Zoomed-out view (10 × observation field) of the image from day 10 in panel e showing the obviously uneven distribution of fibroblasts in coculture-β_1_^-/-^ group. Scale bars, 100 µm. All data are the mean ± SD from 3 independent experiments. Two-way ANOVA and Tukey's multiple comparison test were performed. * *P*<0.05, **** *P*<0.0001, and ns, not significant.

To verify our hypothesis and determine the essential roles of these targets in 3D microstructure formation, we used fibronectin-knockout MEFs (MEF-FN^-/-^), integrin-α_5_-knockout HepG2 (HepG2-α_5_^-/-^) and integrin-β_1_-knockout HepG2 (HepG2-β_1_^-/-^) cells, and labeled them with fluorescent proteins (Fig. S9A-C). Gene-knockout was confirmed by WB analysis (Fig. 3A).

Next, we cocultured different combinations. Unlike coculture-ctrl group in Fig. 3B, all three gene-knockout groups showed obvious interruption of HIIs after day 5, as MEFs and HepG2 cells grew separately without obvious microstructure formation. Fibroblasts gradually died after day 5, probably due to the loss of HIIs stimulation and HepG2 cells as an anchor (Fig. 3C-E). Furthermore, these observations were supported by 2D histograms, as the convergence tendency between two types of pixels was only shown in coculture-ctrl group between day 5 to day 10 (Fig. S9D).

Since fibronectin and collagen I were mainly from fibroblasts, the percentage area occupied by fibroblasts in different groups was quantified. To exclude selection bias as shown in Fig. 3E and Fig. 3H on day 10, three 10 × images from each group were analyzed. Fig. 3F shows that although there is fluctuation, this parameter in coculture-ctrl group remained above 50% beginning on day 2, while for the other groups, this parameter continuously decreased after day 5. On day 10, coculture-FN^-/-^ and coculture-α_5_^-/-^ groups showed a larger decrease than coculture-ctrl group (7.71% and 7.32% vs. 62.82%), while coculture-β_1_^-/-^ group showed a secondary decrease (15.47% vs. 62.82%), which indicated that there were fewer dead fibroblasts in coculture-β_1_^-/-^ group than in the other two gene-knockout groups.

Moreover, as our focus was on HIIs, which would naturally result in the overlap of the two cell types during microstructure formation, the percentage of colocalized areas was then marked and calculated by AutoQuant X3 (Fig. S10A). Fig. 3G shows that all three gene-knockout coculture groups differed significantly from coculture-ctrl group beginning on day 2 in this parameter, which clearly reflected HIIs interruption after gene-knockout.

Next, we tried to explain the observations by WB analysis (Fig. 3A). From results with MEF-FN^-/-^-clover cells, we discovered that fibronectin-knockout not only greatly reduced fibronectin from 1 to 0.01 but also dramatically reduced collagen I from 1 to 0.08. Furthermore, fibronectin-knockout greatly reduced integrin-α_5_ from 1 to 0.29 but had little impact on integrin-β_1_. Similar results were observed in coculture-FN^-/-^ group. From the WB analysis of coculture-α_5_^-/-^ and coculture-β_1_^-/-^ groups, we found that compared with integrin-β_1_, integrin-α_5_ in HepG2 cells had a greater influence on fibronectin expression (0.15 vs. 0.4) and collagen I expression (0.10 vs. 0.56). These results illustrated that integrin-α_5_ had a stronger influence on fibronectin and collagen I expression than integrin-β_1_, which explained why coculture-α_5_^-/-^ group exhibited stronger interference with microstructure formation than coculture-β_1_^-/-^ group. Although integrin-α_5_β_1_ is the only known dimer containing integrin-α_5_, integrin-β_1_ also possesses a complex relationship with other α-class integrins 40, and integrin-β_1_-knockout would inevitably influence other integrins' expression. Indeed, integrin-β_1_-knockout was reported to upregulate other β-class integrins, such as integrin-β_3_ 41-45, which would compensate for its gene-knockout effects. In contrast, integrin-α_5_ is only related to integrin-β_1_, which makes integrin-α_5_ a more specific target than integrin-β_1_ in terms of regulating integrin-α_5_β_1_'s expression.

We also performed IF-staining to observe the distributions of these four proteins in the three gene-knockout coculture groups and found that with the disappearance of microstructures in fibronectin-knockout or integrin-α_5_-knockout coculture groups, IF signals of these proteins were obviously reduced, but in integrin-β_1_-knockout coculture group, the other three proteins weakly clustered around the aggregated fibroblasts (Fig. S10B).

Similar results were obtained in Huh7 and MEFs coculture, after fibronectin-knockout in MEFs or integrin-α_5_-knockout in Huh7 cells (Fig. S11A-D). Collectively, above results indicate that fibronectin from fibroblasts, and integrin-α_5_β_1_ from liver cancer cells are the primary mediators of HIIs during coculture. Additionally, HIIs not only stimulated cancer cell proliferation but also supported fibroblast survival.

### HIIs activated integrin-α_5_β_1_ downstream signaling

**Figure 4:**
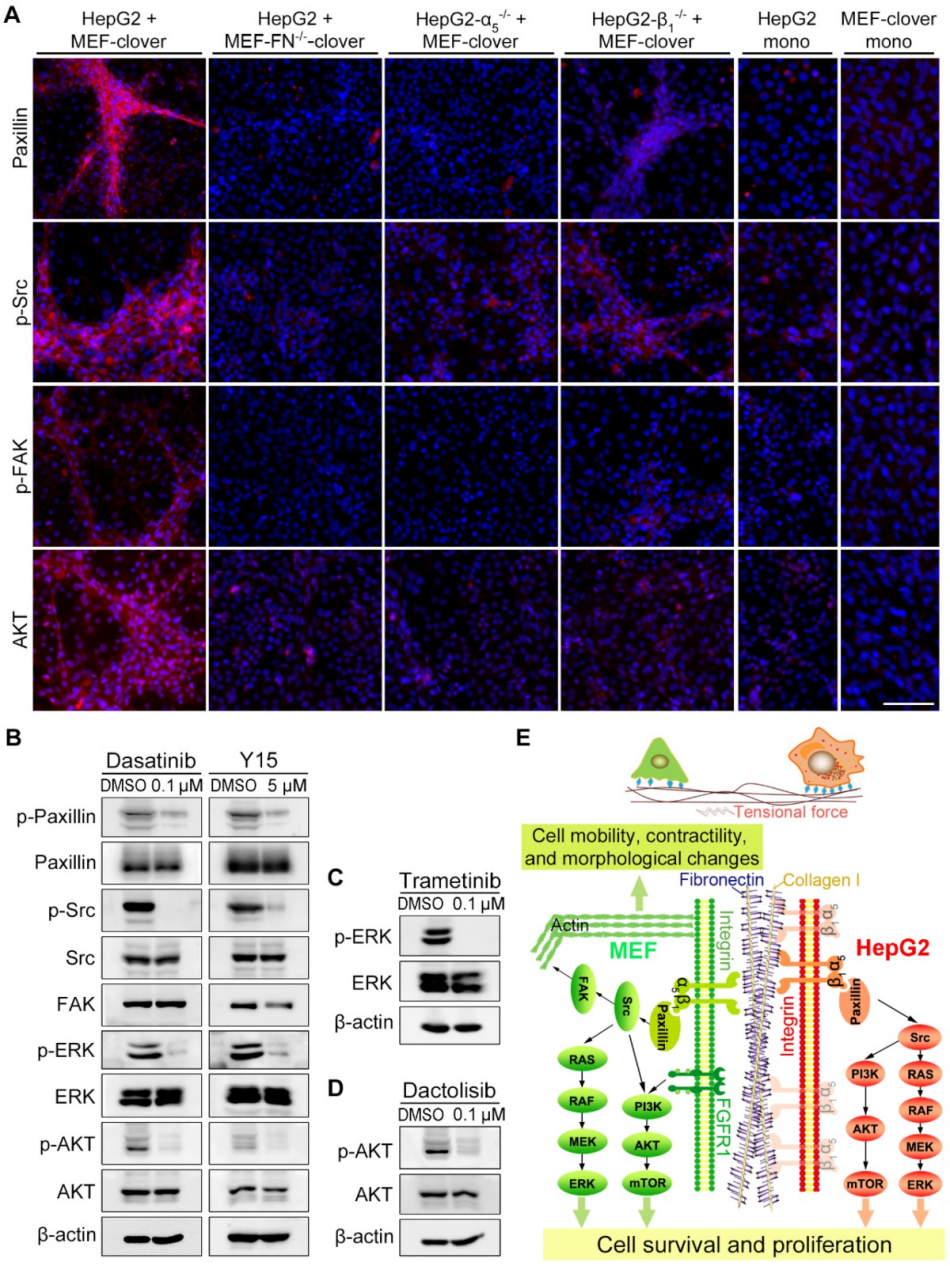
Integrin downstream signaling pathways were activated by fibronectin-integrin-α_5_β_1_-mediated HIIs during coculture. (**A**) Representative IF-staining images on day 10. Nuclei were visualized using Hoechst-33342 staining. Scale bar, 100 µm. (**B**) WB analysis showed that the treatment of coculture-ctrl cells with a FAK inhibitor (Y15, 5 µM) or a Src inhibitor (dasatinib, 0.1 µM) for 10 days reduced p-paxillin, p-Src, p-AKT, and p-ERK1/2 levels. (**C** and **D**) WB results showed that the treatment of coculture-ctrl cells with an MEK inhibitor (trametinib, 0.1 µM) (**C**) or a PI3K inhibitor (dactolisib, 0.1 µM) (**D**) for 10 days reduced p-ERK1/2 or p-AKT levels. (**E**) Diagram depicting heterotypic intercellular interactions (HIIs) between HepG2 cells and MEFs mediated by fibronectin-integrin-α_5_β_1_ and the proposed signaling pathways involved during coculture.

Next, we examined the downstream molecules of integrin-α_5_β_1_ signaling by IF-staining and found that the levels of paxillin, p-Src, p-FAK, and AKT surrounding microstructures formed during the coculture were obviously higher than the adjacent region or monoculture controls (Fig. 4A, Fig. S6, and Fig. S7A). After fibronectin-knockout in MEFs or integrin-α_5_-knockout in HepG2 cells, the above increased signals disappeared with microstructures. However, in the integrin-β_1_-knockout group, weak signals surrounding aggregated MEFs were still observed (Fig. 4A), probably because HIIs was preserved to some extent. Additionally, WB analysis in Fig. S12A showed that downstream signaling molecules, including p-paxillin, p-ERK, and p-AKT, were also affected to a certain extent after gene-knockout in coculture.

To assess the functional involvement of downstream molecules in microstructure formation, we added following inhibitors to coculture system: the FAK inhibitor Y15, the Src inhibitor dasatinib, the MEK inhibitor trametinib, and the PI3K inhibitor dactolisib. WB results showed that the inhibitors obviously blocked the phosphorylation of corresponding targets and their downstream molecules (Fig. 4B-D), resulting in the blockade of microstructure formation, which was similar to the results of gene-knockout cocultures (Fig. S12B vs. Fig. 3C-E). Interestingly, there are indeed some reports on the synergistic application of above inhibitors and other anticancer drugs for the treatment of HCC 46-49, which supported the original intention of our study, i.e., to find key TME-related targets for the combination targeted therapy. Collectively, we confirmed that the integrin-FAK-Src, MEK-ERK, and PI3K-AKT pathways were involved in microstructure formation.

Additionally, the effect of stretch forces on HepG2 cells was observed by IF-staining using a human-specific integrin-α_5_β_1_ antibody, which clearly illustrated morphological changes in certain HepG2 cells in coculture (from a typical round shape to a long, unidirectionally patterned shape) (Fig. S12C). The colocalization of fibronectin with integrin-α_5_/integrin-β_1_ was also verified (Fig. S12D and E). In Fig. 4E, we summarized the HIIs mechanism and signaling pathways during coculture.

### HIIs-primed liver cancer cells displayed higher tumorigenic ability

**Figure 5:**
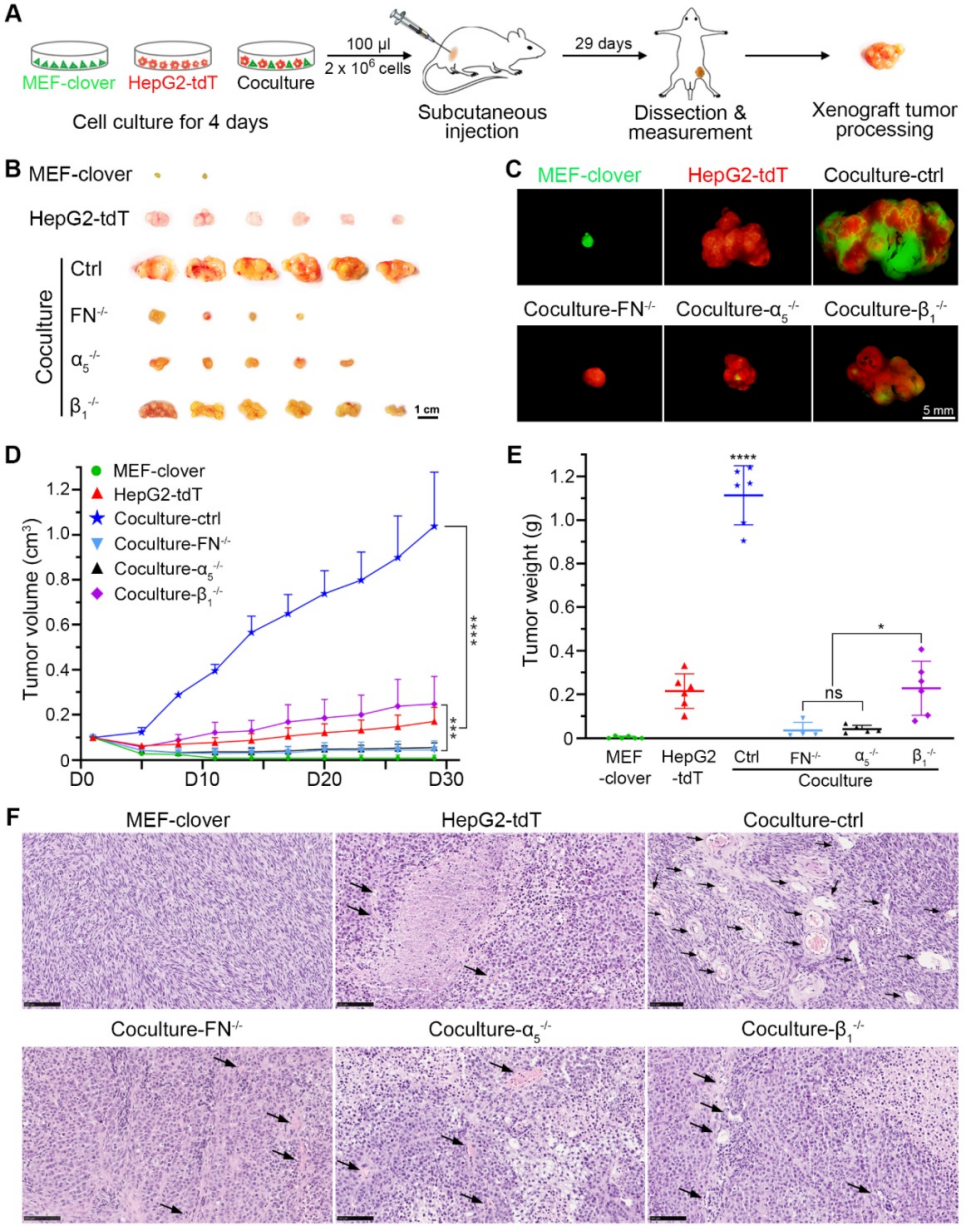
HIIs-primed HepG2 cells exhibited greater tumor-formation ability than the controls. (**A**) Schematic of the procedure used to establish the HCC xenograft nude mouse model. Cells in each group were cultured for 4 days before subcutaneous injections. For each mouse, 2 × 10^6^ cells in 100 µl were injected. (**B**) Representative bright-field images of xenografts in different groups on day 29. Scale bar, 1 cm. (**C**) Representative fluorescent images of xenografts in different groups on day 29. Scale bar, 5 mm. (**D**) Quantification of the xenograft tumor volume in different groups. The tumor volume data were obtained every 3 days from day 5 to day 29 (tumor volume was assigned to 0.1 cm^3^ on day 1). Two-way ANOVA and Tukey's multiple comparison test were performed. (**E**) Quantification of the xenograft tumor weight in different groups. The tumor weight data were obtained on day 29 after sacrifice and dissection. One-way ANOVA and Tukey's multiple comparisons test were performed. (**F**) Representative H&E-stained images showing the abundance of blood vessels inside the tumor xenografts in different groups. Scale bars, 100 µm. All data were obtained from 6 mice in each group and are represented as the mean ± SD. * *P*<0.05, *** *P*<0.001, **** *P*<0.0001.

To determine the situation *in vivo*, we established a xenograft tumor model in nude mice as shown in Fig. 5A. The xenograft volume of each mouse was monitored every 3 days until day 29. The xenografts (Fig. 5B-E) show that although MEFs have no tumorigenic ability, they helped HepG2 cells form larger tumors, with the tumor volume and tumor weight in coculture-ctrl group increasing by 6.0-fold and 5.2-fold, respectively, compared with monocultured HepG2 group. These data indicated that HepG2 cells primed with HIIs exhibited greater tumor formation and tumor growth ability, which were significantly inhibited in gene-knockout groups, with tumor volume and tumor weight in all three groups decreasing by more than 75% and 79%, respectively. Furthermore, fibronectin-knockout and integrin-α_5_-knockout had a better inhibitory effect than integrin-β_1_-knockout (Fig. 5B-E). A more significant difference was found using Huh7 and MEFs, in which monocultured Huh7 cell failed to grow into xenografts tumors, while cocultured Huh7 cells grew into tumors with a mean volume of 0.18 cm^3^ or a mean weight of 0.15 g (Fig. S13A-C).

Based on above results, we further hypothesized that the vascular density in coculture-ctrl group was higher than that of the other groups. Next, hematoxylin and eosin (H&E) staining was performed using paraffin-embedded sections, and phase images were captured and used to compare vascular abundance between different groups. As shown in Fig. 5F, coculture-ctrl groups had 6-fold more vessel-like structures containing red blood cells per 20 × observation field than monocultured groups. Moreover, this parameter was greatly decreased in all three gene-knockout groups. Collectively, these results suggested the potential involvement of HIIs in pathological angiogenesis.

### Fibronectin and collagen I remodeling induced by HIIs promoted HUVEC alignment and elongation

Based on above *in vivo* experiments, we introduced HUVECs into our coculture system to form a triculture system. To visualize HUVECs, we first labeled them with a near-infrared fluorescent protein (iRFP), iRFP-670 (i670), and installed a suitable filter to exclude bleed-through. We then tricultured three cell types and found that the 5:3:2 ratio produced the best outcome on day 10, as HUVECs had the best differentiation (Fig. S14A).

**Figure 6:**
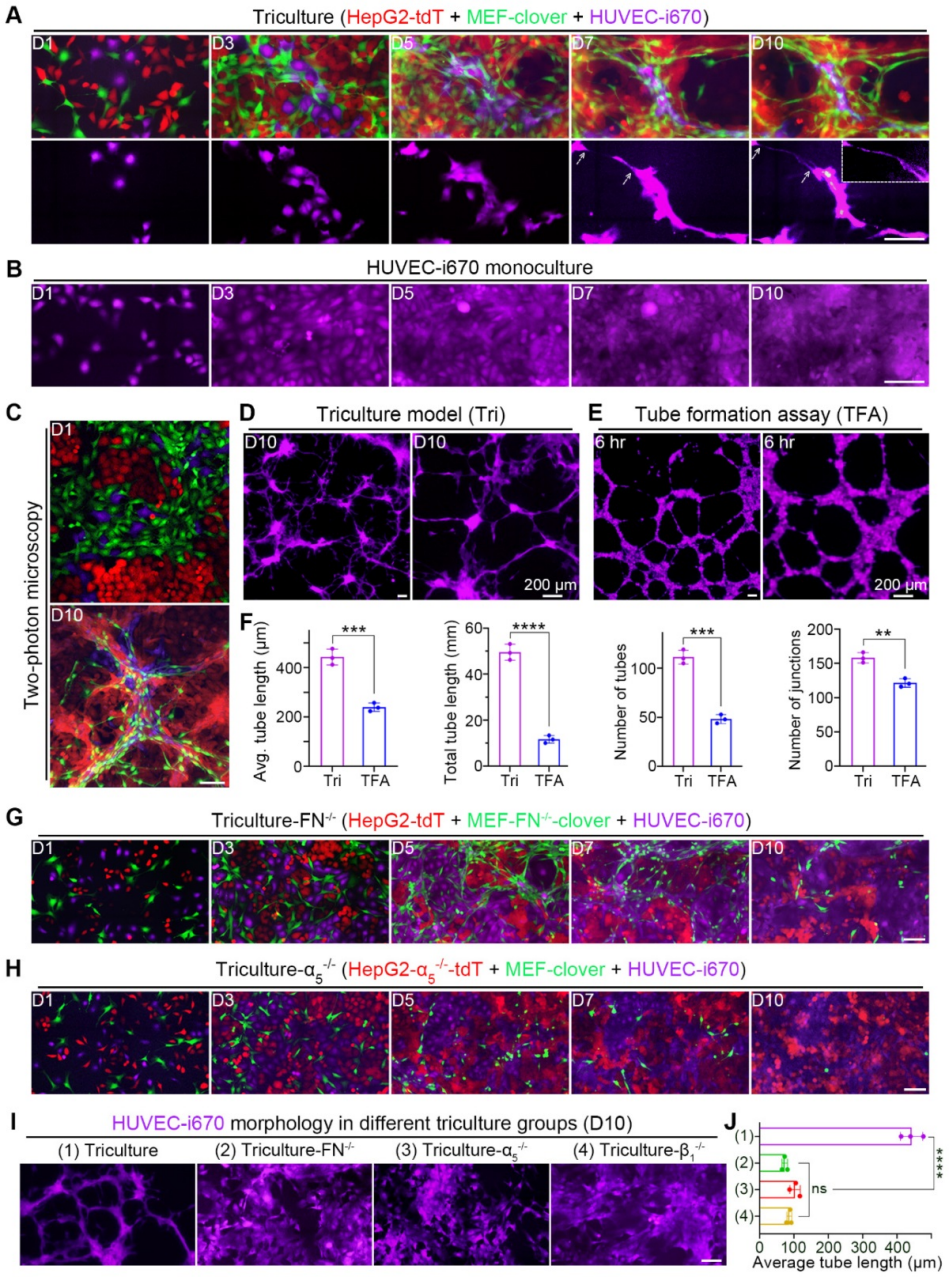
HUVECs aligned to and elongated along microstructures in triculture. (**A**) Representative images showing tricultured cells at the same spots from day 1 to day 10. Corresponding HUVEC-i670 morphology is listed below. Zoomed-in image in the right-upper corner on day 10 showing the small tube. Scale bar, 100 µm. (**B**) Representative images of monocultured HUVEC-i670 cells at the same spots from day 1 to day 10. Scale bar, 100 µm. (**C**) Representative two-photon images of tricultured cells on day 1 and day 10. Scale bar, 100 µm. (**D** and **E**) Representative zoomed-out and zoomed-in images of HUVEC-i670 in triculture on day 10 (**D**) or HUVEC-i670 after incubation on Matrigel for 6 hr (**E**). Scale bars, 200 µm. (**F**) Angiogenic parameters of HUVEC-i670 cells in triculture on day 10 (left) and tube formation assay (TFA) at 6 hr (right). Values were quantified over 5 × observation fields with AngioTool software. Unpaired t test was performed. (**G** and **H**) Representative images of the following triculture groups from day 1 to day 10: triculture-FN^-/-^ (**G**) and triculture-α_5_^-/-^ (**H**). Scale bar, 100 µm. (**I**) Representative images showing HUVEC-i670 morphology in different triculture groups (for triculture-β_1_^-/-^ group, please refer to Fig. S9E). Scale bar, 100 µm. (**J**) Quantification of the average tube length of HUVECs in different triculture groups on day 10. Values were quantified over 10 × observation fields. One-way ANOVA was performed. All data are the mean ± SD from 3 independent experiments. ** *P*<0.01, *** *P*<0.001, **** *P*<0.0001, and ns, not significant.

Chronological fluorescent images taken at the same spots showed that compared with monocultured HUVECs in Fig. 6B, HUVECs in triculture underwent dramatic morphological changes; not only was the cell body elongated, but long and thin branches extended (Fig. 6A). Interestingly, HUVECs aligned with 3D microstructures and colocalized with MEFs, as certain extended branches perfectly colocalized with elongated MEFs (Fig. S14B), suggesting the role of fibroblasts in HUVEC migration and differentiation. Additionally, 3D volume-rendering images obtained by two-photon microscopy are shown in Fig. 6C, which provides us with a more comprehensive view. Similar results were obtained in the triculture of Huh7 cells (Fig. S15A and B).

Since no obvious differentiation was observed in monocultured HUVECs (Fig. 6B), we compared tricultured HUVECs with HUVECs in a classical tube-formation assay (TFA) (Fig. 6D-E). After quantification using AngioTool software, features of angiogenesis (the length, number and junction of the tubes from HUVECs) were compared. From the results, we observed that HUVEC differentiation in triculture was better than that in TFA, with more and longer branches (Fig. 6F).

Furthermore, the coculture results in Fig. S14C and D suggested that HUVEC differentiation required HIIs between HepG2 cells and MEFs. To verify this assumption, we further performed triculture using gene-knockout cell types to interrupt HIIs. As expected, the patterned alignment and elongation of HUVECs were interrupted with the disappearance of microstructures in these gene-knockout triculture groups, leaving HUVECs to develop into colonies (Fig. 6G and H, Fig. S14E). To compare the morphology of HUVECs in these groups with that of HUVECs in normal triculture group, far-red channel images of all groups were collectively shown in Fig. 6I and then analyzed using AngioTool software. The average tube length of HUVECs in these groups was used to illustrate the effects of disrupting HIIs between HepG2 cells and MEFs on HUVEC differentiation (Fig. 6J). From these results, we observed that HUVEC elongation was significantly reduced after gene-knockout, with the average tube length decreasing from 442.3 μm to below 100 μm in all gene-knockout triculture groups. These results confirmed that the alignment and elongation of HUVECs required HIIs between liver cancer cells and MEFs.

### HUVEC alignment and elongation in triculture were dependent on integrin-α_5_ and integrin-β_1_ and facilitated by ECM-bound VEGFA secreted by MEFs

Based on above results, we further explored how HIIs provide the “route” and guide HUVEC. A VEGFA concentration gradient was reported to be involved 50-52. Next, we performed q-PCR using species-specific primers to quantify VEGFA mRNA changes during coculture, and found that the mRNA level was increased by 69.4% in cocultured MEFs compared with monocultured MEFs on day 10 (Fig. S16A). To confirm these results, we measured mouse VEGFA (mVEGFA) levels in conditioned medium (CM) by a mouse-specific ELISA kit. The levels of cumulative mVEGFA in the medium of coculture and triculture on day 3 were increased by more than 2-fold compared with those of monocultured MEFs (Fig. S16B). Given that ECM-bound VEGFA is reported more potent than free VEGFA 52-54, evidence of ECM-bound VEGFA would lay a solid basis for the above unknown questions of how HIIs provide the “route” and guide HUVECs. Additionally, as the primary VEGFA mediator, VEGFR2 activation could promote endothelial cell differentiation and proliferation 55-57.

**Figure 7:**
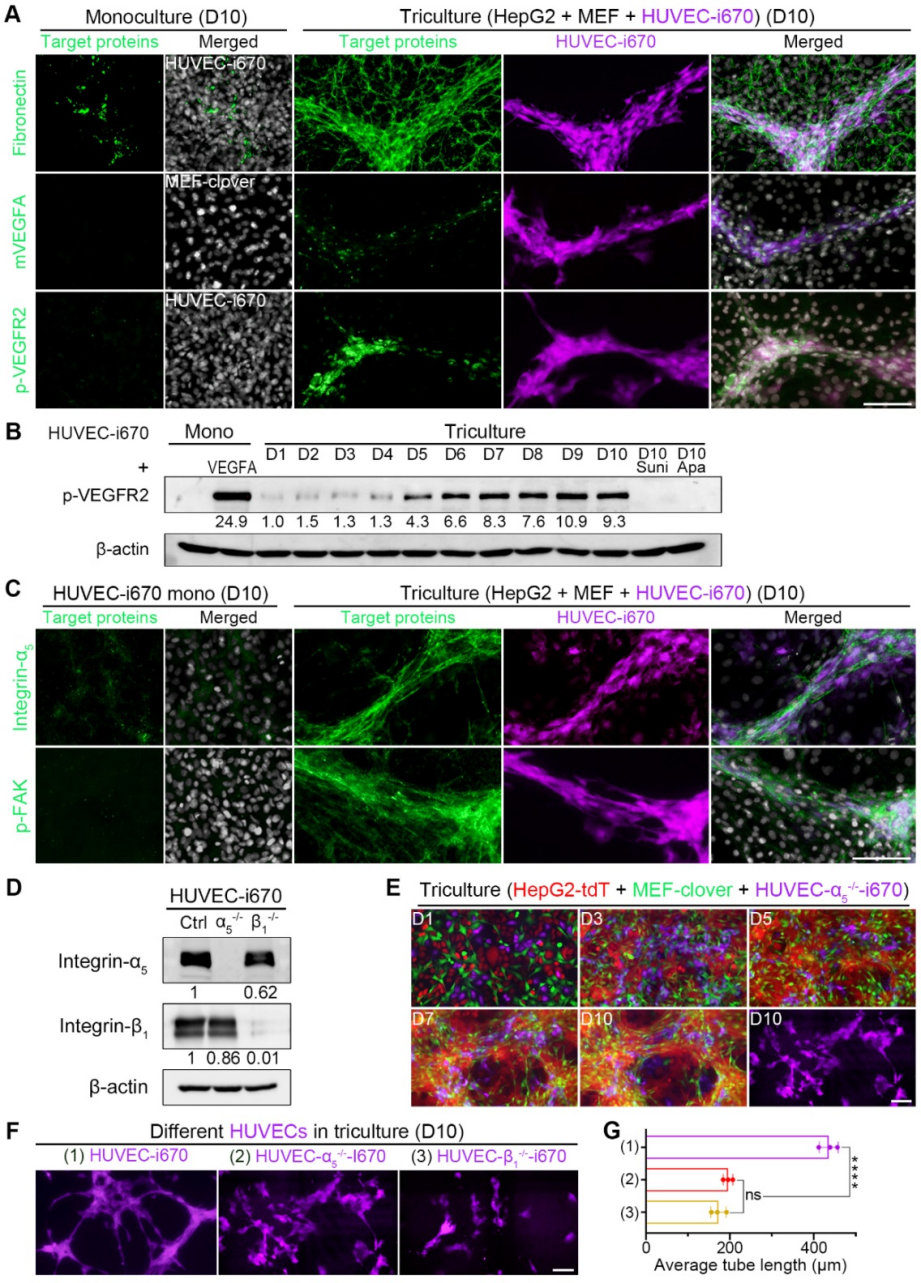
HUVEC migration to 3D multilayer microstructures enriched with ECM-bound VEGFA required integrin-α_5_β_1_. (**A**) Representative IF-staining images. (**B**) WB results showing the levels of p-VEGFR2 in monocultured HUVEC-i670 on day 10, the positive control (monocultured HUVEC-i670 with 25 ng/ml rhVEGFA treatment for 5 min), tricultured cells from day 1 to day 10, and the tricultured cells on day 10 administrated with sunitinib (10 nM) or apatinib (10 nM) for 10 days. Drugs were added to the medium beginning on day 2, and the medium was changed daily. (**C**) Representative IF-staining images. (**D**) WB analysis showed the levels of integrin-α_5_ and integrin-β_1_ in HUVEC-i670, HUVEC-α_5_^-/-^-i670, and HUVEC-β_1_^-/-^-i670 cells. (**E**) Representative fluorescence live-cell images at the same spots. The far-red channel in the right-lower corner shows HUVEC-α_5_^-/-^-i670 morphology in triculture on day 10. (**F**) Representative fluorescent images of the following types of HUVECs in triculture with HepG2-tdT and MEF-clover cells on day 10: HUVECs-i670, HUVECs-α_5_^-/-^-i670, and HUVECs-β_1_^-/-^-i670 (please refer to Fig. S11C). (**G**) Quantification of the average tube length of the above types of tricultured HUVECs on day 10. Values were quantified over a 10 × observation field from 3 independent experiments. Data are mean ± SD. One-way ANOVA and Tukey's multiple comparisons test were performed. **** *P*<0.0001, and ns, not significant. Scale bars, 100 µm.

Based on above results and analysis, we then performed IF-staining of related targets in triculture cells on day 10. From Fig. 7A, Fig. S16C, and Fig. S16D, we observed that HUVECs colocalized with fibronectin, collagen I, and matrix-bound mVEGFA during triculture. The obviously elevated p-VEGFR2 IF signals were supported by WB analysis showing an obvious upregulation of p-VEGFR2 level in tricultured HUVECs after day 5, with the level on day 10 comparable to that of monocultured HUVECs treated with 25 ng/ml VEGFA for 5 min (Fig. 7B). Furthermore, we applied clinically approved small-molecule drugs targeting VEGFR2 to triculture system. The administration of either sunitinib or apatinib at a concentration of 10 nM for 10 days almost completely abolished VEGFR2 phosphorylation (Fig. 7B) and greatly inhibited HUVEC differentiation, with the average tube length decreasing significantly from 442.3 μm to 65.7 μm or 92.3 μm, respectively (Fig. S16E and F). These results indicated the potential application of our triculture system in screening antiangiogenic agents for HCC treatment.

Since integrin-α_5_β_1_ can mediate cell attachment and migration on fibronectin 58-61, we next explored whether HUVEC migration and differentiation utilized integrin-α_5_β_1_. From IF-staining results, we found that the enhanced integrin-α_5_, integrin-β_1_, and p-FAK signals colocalized with HUVEC distribution in 3D microstructures (Fig. 7C, Fig. S16C and D), suggesting the involvement of integrin-α_5_ and integrin-β_1_ in HUVEC migration, which activated integrin-mediated downstream signaling. To verify this hypothesis, we generated integrin-α_5_-knockout HUVECs (HUVEC-α_5_^-/-^) or integrin-β_1_-knockout HUVECs (HUVEC-β_1_^-/-^) (Fig. 7D, Fig. S17A and B). The migration of these two cell types to 3D microstructures was interrupted to a certain extent when tricultured with HepG2-tdT and MEF-clover cells (Fig. 7E and Fig. S17C). More importantly, when we compared the far-red channel images on day 10 from these two groups with those of the normal triculture group (Fig. 7F), we observed that HUVEC differentiation was also affected. After analysis with AngioTool software, we found that the average tube length decreased by more than 55% in the two groups (Fig. 7G), which illustrated that HUVEC migration and elongation required integrin-α_5_ and integrin-β_1_ in HUVECs and further highlighted the role of 3D microstructures in HUVEC differentiation. Apart from the apparent reason that their migration was affected after gene-knockout, another reason might be that the cross-activation between VEGFR2 and integrins was also affected 62-64.

### Clinical relevance of fibronectin, collagen I, integrin-α_5_, and integrin-β_1_ and their downstream signaling molecules in HCC

To assess the clinical significance of fibronectin, collagen I, integrin-α_5_, integrin-β_1_ and their downstream signaling molecules in HCC, we performed IHC-staining in 52 HCC tumors and paired adjacent nontumorous tissues. The main characteristics of these 52 patients with HCC were summarized in Table S1.

**Figure 8:**
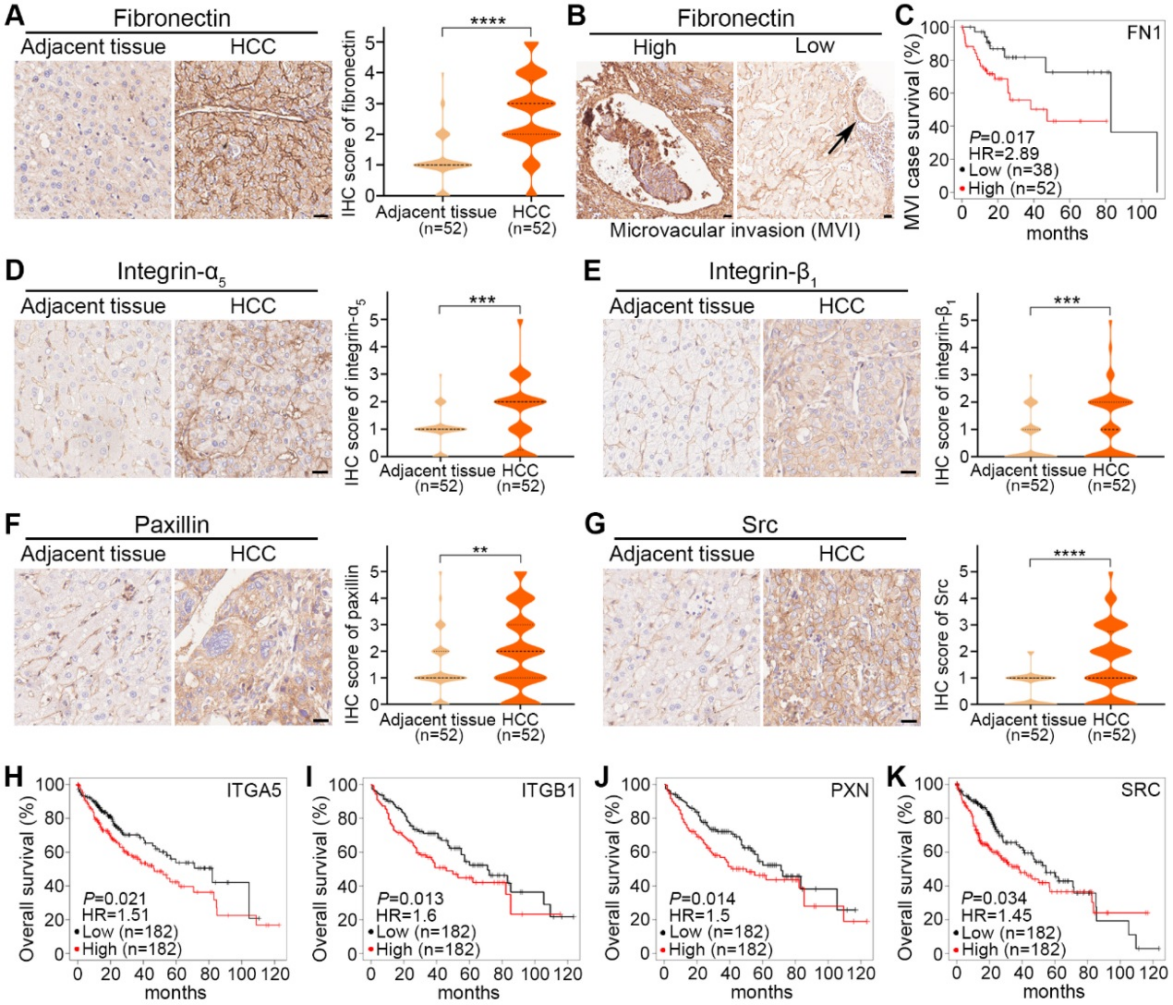
Clinical significance of fibronectin, integrin-α_5_, integrin-β_1_ and their downstream signaling molecules in HCC tumors. (**A**) Representative IHC-staining images of fibronectin expression in adjacent tissues and HCC tumors from HCC patients (left) and violin plots of IHC scores from these 52 patients (right). (**B**) Representative images of microvascular invasion (MVI) events found by IHC-staining for fibronectin with high (≥3) and low (≤2) IHC scores. (**C**) Correlation of FN1 mRNA levels with the survival rate of HCC patients with MVI events in the KMplot dataset. (**D** to **G**) Representative IHC-staining images of integrin-α_5_ expression (**D**), integrin-β_1_ expression (**E**), paxillin expression (**F**), and Src expression (**G**) in adjacent tissues and HCC tumors from HCC patients (left), and violin plots of IHC scores from these 52 patients (right). Scale bars, 20 µm. Data are shown as medians and quartiles. Unpaired t test was performed. HR, hazard ratio. ** *P*<0.01, *** *P*<0.001, **** *P*<0.0001, and ns, not significant. (**H** to **K**) Correlation of ITGA5 (**H**), ITGB1 (**I**), PXN (**J**), and SRC (**K**) mRNA levels with the overall survival rate of HCC patients in the KMplot dataset.

As shown in representative images and quantified results, fibronectin expression was significantly higher in HCC tumors than in adjacent tissues (Fig. 8A). To investigate the clinical significance of fibronectin in HCC, we classified these 52 patients into 2 groups: fibronectin high (IHC score≥3) and low (IHC score≤2), and analyzed the association between fibronectin expression status and certain clinicopathological characteristics. The results indicated that fibronectin high expression was closely correlated with microvascular invasion (MVI), tumor size, and TNM stages (Table 1). We also observed several MVI cases in our IHC result analysis (Fig. 8B), and more importantly, higher mRNA levels of FN1 genes were found corresponding to a shorter survival time in HCC cases with MVI in the KMplot dataset (Fig. 8C). However, no significant difference was found in collagen I (Fig. S17D). The results suggested that fibronectin might be a better marker to indicate MVI or HCC metastasis than collagen I.

For integrin-α_5_, integrin-β_1_, paxillin, or Src, similar increased expression in HCC tumors compared with adjacent tissues is shown in representative images and quantified results (Fig. 8D-G). Additionally, a shorter survival time was correlated with higher mRNA levels of ITGA5, ITGB1, PXN, or SRC in the KMplot dataset obtained from HCC patients (Fig. 8H-K). No significant difference in FAK was found (Fig. S17E).

## Discussion

The development of nonsurgical therapies for HCC is relatively slow compared with those for other cancers, largely due to the presence of cirrhosis or other chronic liver diseases, which provides a constant niche suitable for the maintenance of cancer cell stemness and the subsequent recurrence 8, 65. Moreover, the localization of secondary tumors also seems to be orchestrated by the TME 11. Hence, understanding the HIIs between liver cancer cells and major stromal cells in the TME, such as fibroblasts and endothelial cells, is of great importance for the development of combination targeted therapies.

The best way to study HIIs between cancer cells and stromal cells is through coculture. However, due to the difficulties in separating different cell types after coculture, many studies avoided direct coculture 66-69. Here, we tried to solve this issue by coculturing liver cancer cells from humans with fibroblasts from mice, and then exploited the difference between them to detect the protein or mRNA level changes of targets in either cell type after coculture. Moreover, unlike previous studies, we extended coculture time to 10 days to fully examine HIIs and discovered microstructure formation. Additionally, we established a direct triculture system for HCC and discovered the effects of microstructures on angiogenesis induction. Furthermore, we found that in addition to serving as a receptor, integrin-α_5_β_1_ could mediate HIIs and remodel fibronectin and collagen I, which became the foundation for tumorous structure formation. Although the mechanism of ECM remodeling and the many roles of remodeled ECM have been well studied 70-73, the functions of the remodeled ECM in microstructure formation and angiogenesis have not been reported. Finally, we first reported that HUVECs could migrate and differentiate along microstructures containing ECM-bound VEGFA, which is dependent on integrin-α_5_β_1_ in HUVECs.

Nevertheless, there are also some shortcomings in this study. First, as mentioned above, it is very difficult to separate fibroblasts from liver cancer cells after coculture, and commercially available species-specific antibodies are few, which makes performing related experiments inconvenient. Second, it takes 10 days for our direct coculture and triculture systems to be established, which is rather time-consuming compared with others models, but we believe that our systems are closer to the natural process of tumor formation in animal models. Moreover, the generation of purified gene-knockout cell lines in this study was rather time-consuming.

## Conclusion

In summary, this study explored HIIs in HCC and revealed that HIIs mediated by fibronectin and integrin-α_5_β_1_ could promote 3D growth of cancer cells by forming 3D microstructures. In this process, fibronectin and collagen I were remodeled by upregulated integrins in cancer cells, and VEGFA was enriched in remodeled ECM and induced endothelial cell migration and differentiation. Furthermore, the expression level of fibronectin, integrin-α_5_ and integrin-β_1_ were higher in 52 HCC tumors than in paired adjacent tissues. These findings lay a solid basis for the future development of combination targeted therapies for HCC.

## Supplementary Material

Supplementary figures and tables.

## Figures and Tables

**Table 1:** Correlation analysis of fibronectin expression with clinicopathological characteristics

Variables	IHC scores of fibronectin (n)		*P* value
	High (≥3)	Low (≤2)	
**Microvascular invasion**			
No	5	28	*P*<0.01
Yes	11	8	
**Tumor size**			
≤5 cm	3	23	*P*<0.01
>5 cm	13	13	
**TNM stage**			
I	10	23	*P*<0.05
II	4	1	
III	2	12	

Pearson's chi squared test was used.
