# Supplementary material for: The interactions between integrin α5β1 of liver cancer cells and fibronectin of fibroblasts promote tumor growth and angiogenesis
Source: Int J Biol Sci. 2022 Aug 1;18(13):5019–37. doi: 10.7150/ijbs.72367 (PMC9379399; doi:10.7150/ijbs.72367)
Supplement: Supplementary file 1 — Supplementary figures and tables. [file ijbsv18p5019s1.pdf]

Supplementary materials

The interactions between integrin $\alpha_5\beta_1$ of liver cancer cells and fibronectin of fibroblasts promote tumor growth and angiogenesis

Zheng Peng[1], Meng Hao[1], Haibo Tong[1], Hongmei Yang[1], Bin Huang[1], Zhigang Zhang[2], Kathy Qian Luo[1,3]*

[1] Faculty of Health Sciences, University of Macau, Taipa, Macao SAR, China

[2] State Key Laboratory of Oncogenes and Related Genes, Shanghai Cancer Institute, Renji Hospital, School of Medicine, Shanghai Jiao Tong University, Shanghai, China

[3] Ministry of Education-Frontiers Science Center for Precision Oncology, University of Macau, Taipa, Macao SAR, China

* Corresponding author: Kathy Qian Luo, Faculty of Health Sciences, University of Macau, Taipa, Macao SAR, China. Tel: 853-8822-4233; Fax: 853-8822-2314; Email address: kluo@um.edu.mo

Supplementary figures

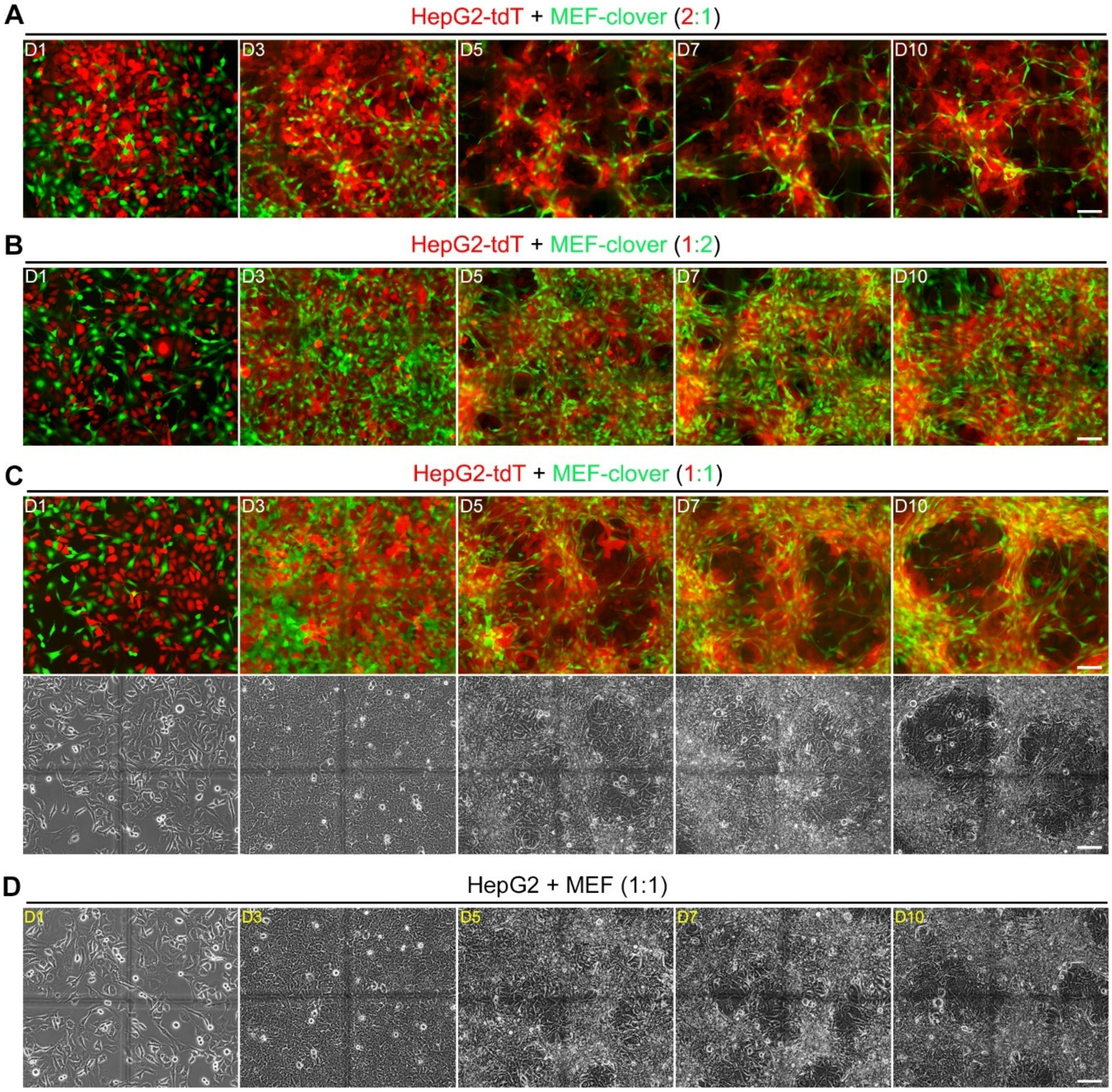

Fig. S1. The best ratio of HepG2-tdT and MEF-clover cells for coculture was 1 to 1. (**A** and **B**) Representative live-cell fluorescence images showing the coculture of HepG2-tdT and MEF-clover cells at ratios of 2:1 (**A**) and 1:2 (**B**) at the same spots from day 1 to day 10. (**C**) Representative live-cell fluorescence images showing the coculture of HepG2-tdT and MEF-clover cells at a ratio of 1:1 at the same spots from day 1 to day 10, and corresponding phase images are also listed below. (**D**) Representative live-cell phase images showing the coculture of HepG2 and MEFs at a ratio of 1:1 at the same spots from day 1 to day 10. All photographs were taken on a Zeiss Axio Observer microscope. Scale bars, 100 µm.

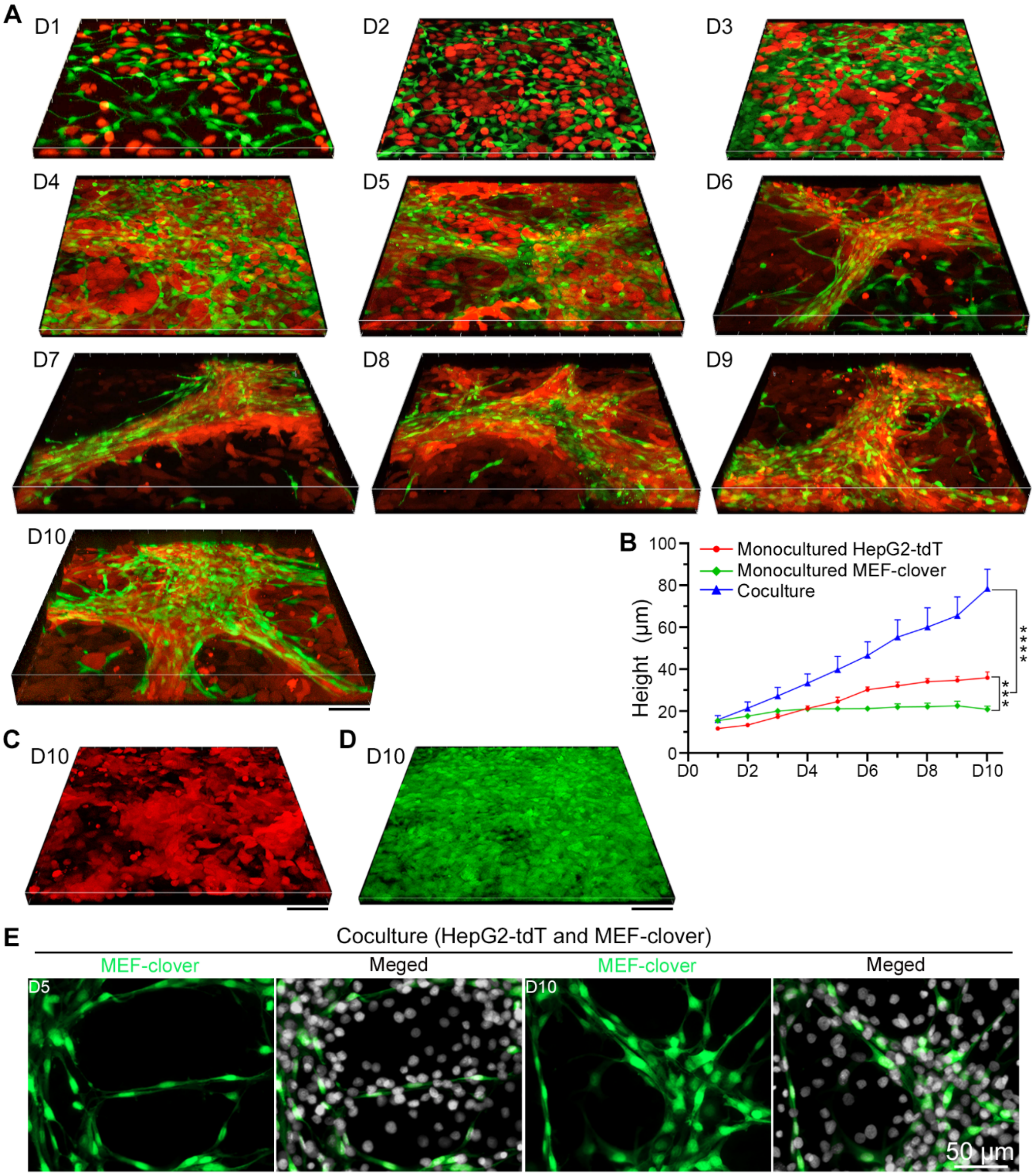

Fig. S2. Elongated MEF-clover cells were mainly distributed in 3D multilayer microstructures. (**A**) 3D volume-rendering images showing the formation and increasing height of 3D multilayer microstructures during the coculture of HepG2-tdT and MEF-clover cells. Scale bar, 100 μm. (**B**) Plot showing changes in the height of monocultured MEF-clover cells, monocultured HepG2-tdT cells and their coculture from day 1 to day 10. Values were quantified from 3 independent experiments and are shown as the mean ± SD. Two-way ANOVA and Tukey's multiple comparison test were performed. *** $P<0.001$, **** $P<0.0001$. (**C** and **D**) 3D volume-rendering images showing monocultured HepG2-tdT (**C**) and MEF-clover cells (**D**) on day 10. Scale bars, 100 μm. (**E**) Representative fluorescent images showing the morphology of MEF-clover cells in coculture on day 5 and day 10 after fixation. Nuclei were visualized using Hoechst-33342 staining. Scale bar, 50 μm.

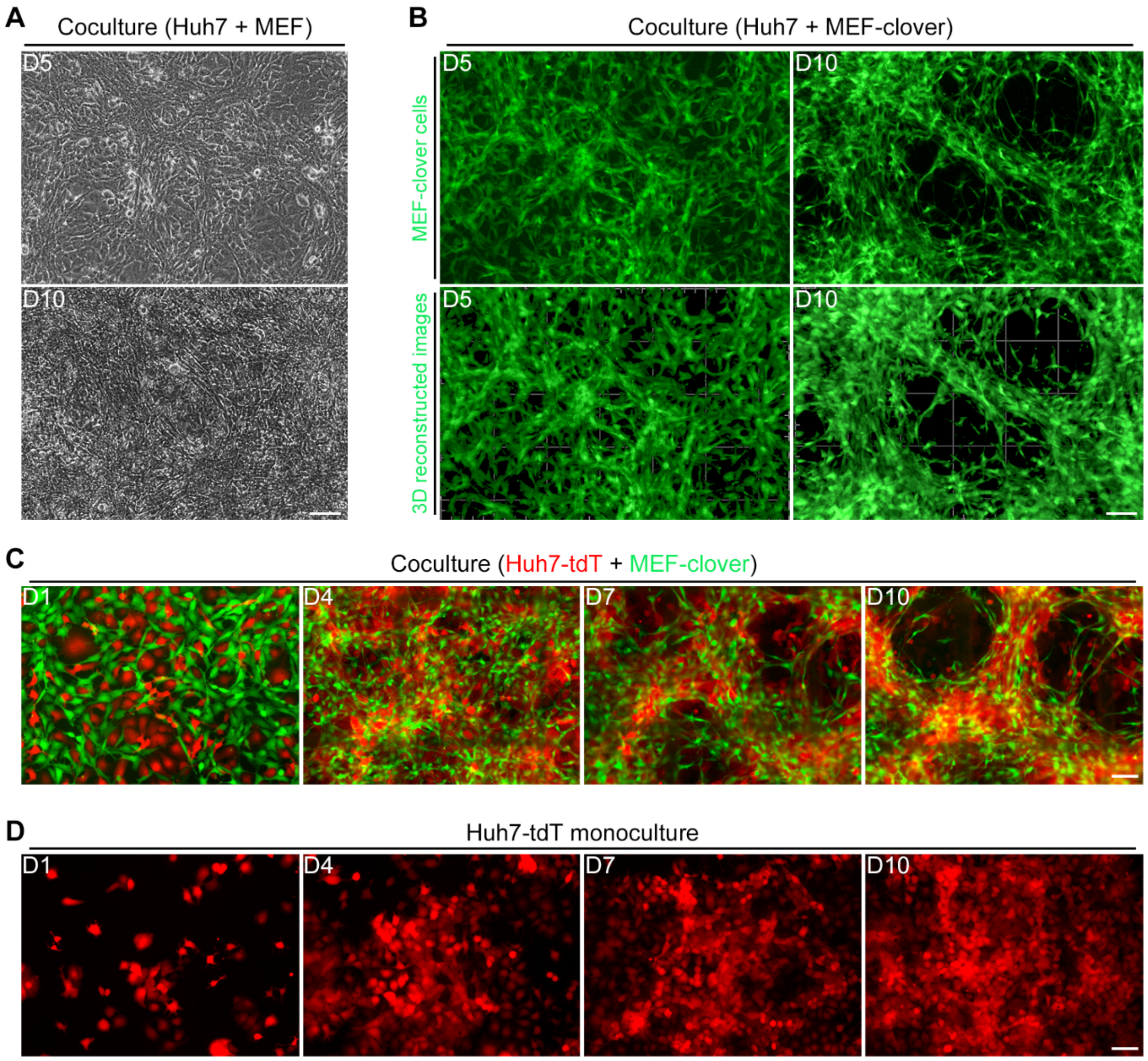

Fig. S3. Coculture of Huh7 cells and MEFs led to the formation of 3D multilayer microstructures. (**A**) Representative live-cell phase images showing the coculture of Huh7 and MEFs at a ratio of 1:1 on day 5 and day 10. (**B**) Representative live-cell fluorescence images and 3D reconstructed images showing the morphology and distribution of MEF-clover cells during coculture (Huh7 and MEF-clover cells) on day 5 and day 10. (**C**) Representative live-cell fluorescence images demonstrating the formation of 3D multilayer microstructures at the same spot from day 1 to day 10. (**D**) Representative fluorescence images of monocultured Huh7-tdT cells at the same spot. Scale bars, 100 μm.

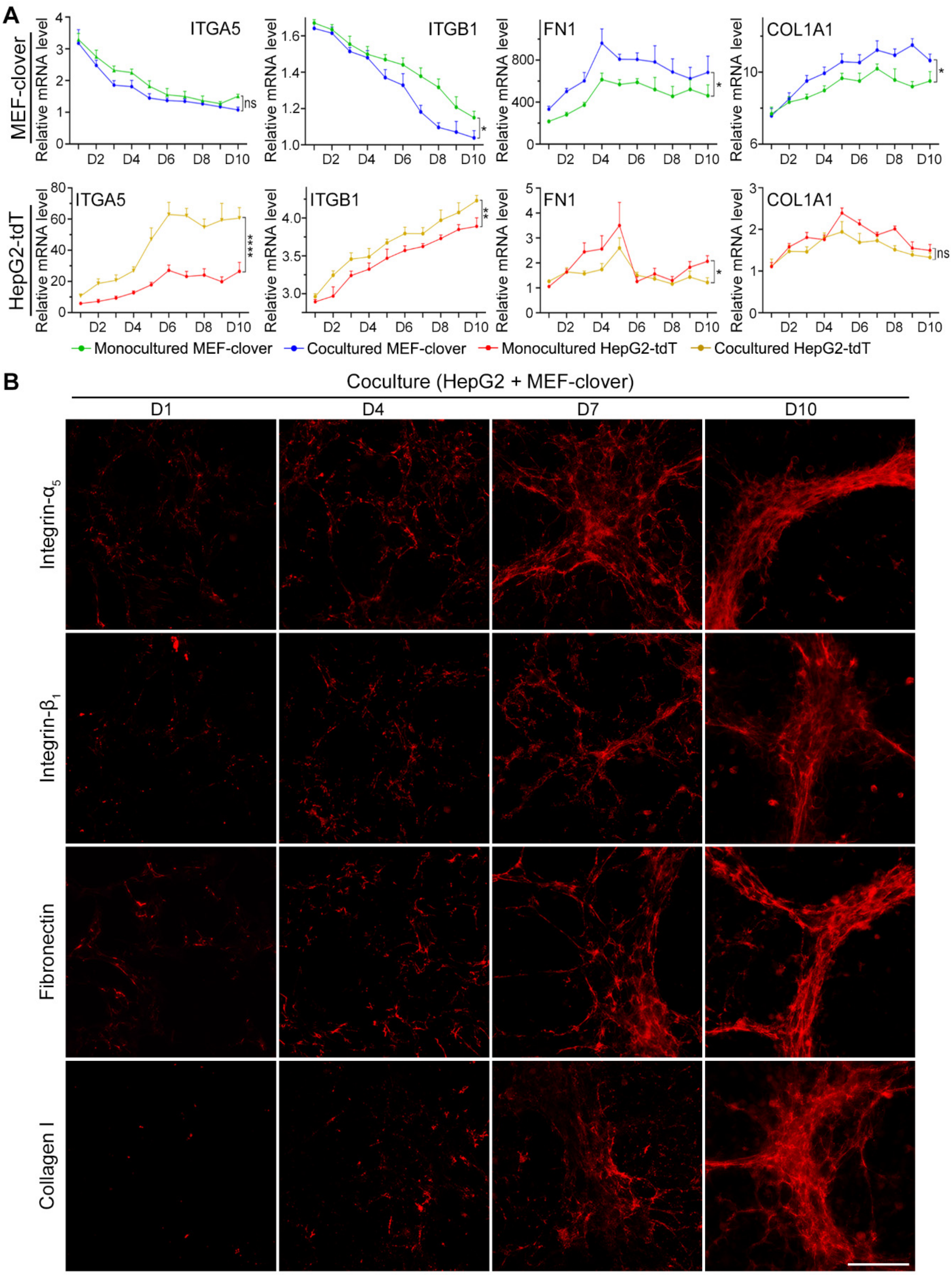

Fig. S4. Integrin-α_5, integrin-β_1, fibronectin, and collagen I displayed significantly upregulated expression during coculture. (**A**) Relative mRNA levels of ITGA5, ITGB1, FN1, and COL1A1 were analyzed using species-specific primers (please refer to the Material and methods section). For monocultured and cocultured MEF-clover cells, mRNA levels were analyzed using primers that recognize only the corresponding mRNA from mice. For monocultured and cocultured HepG2-tdT cells, mRNA levels were analyzed using primers that recognize only the

corresponding mRNA from humans. The final results were normalized to the same common GAPDH primers suitable for humans and mice. Values were quantified from 3 independent experiments and are shown as the mean ± SD. Two-way ANOVA and Tukey's multiple comparison test were performed. * $P<0.05$, ** $P<0.01$, **** $P<0.0001$, and ns, not significant. (**B**) Time-course IF-staining showing changes in the structure and distribution of related proteins. HepG2 and MEF-clover cells were mixed at a ratio of 1:1 and seeded on coverslips in 6-well plates on day 0. Then, the coverslips were harvested on day 1, day 4, day 7, and day 10. Fluorescent images were taken on a Zeiss Axio Observer microscope after IF-staining. Scale bar, 100 μm.

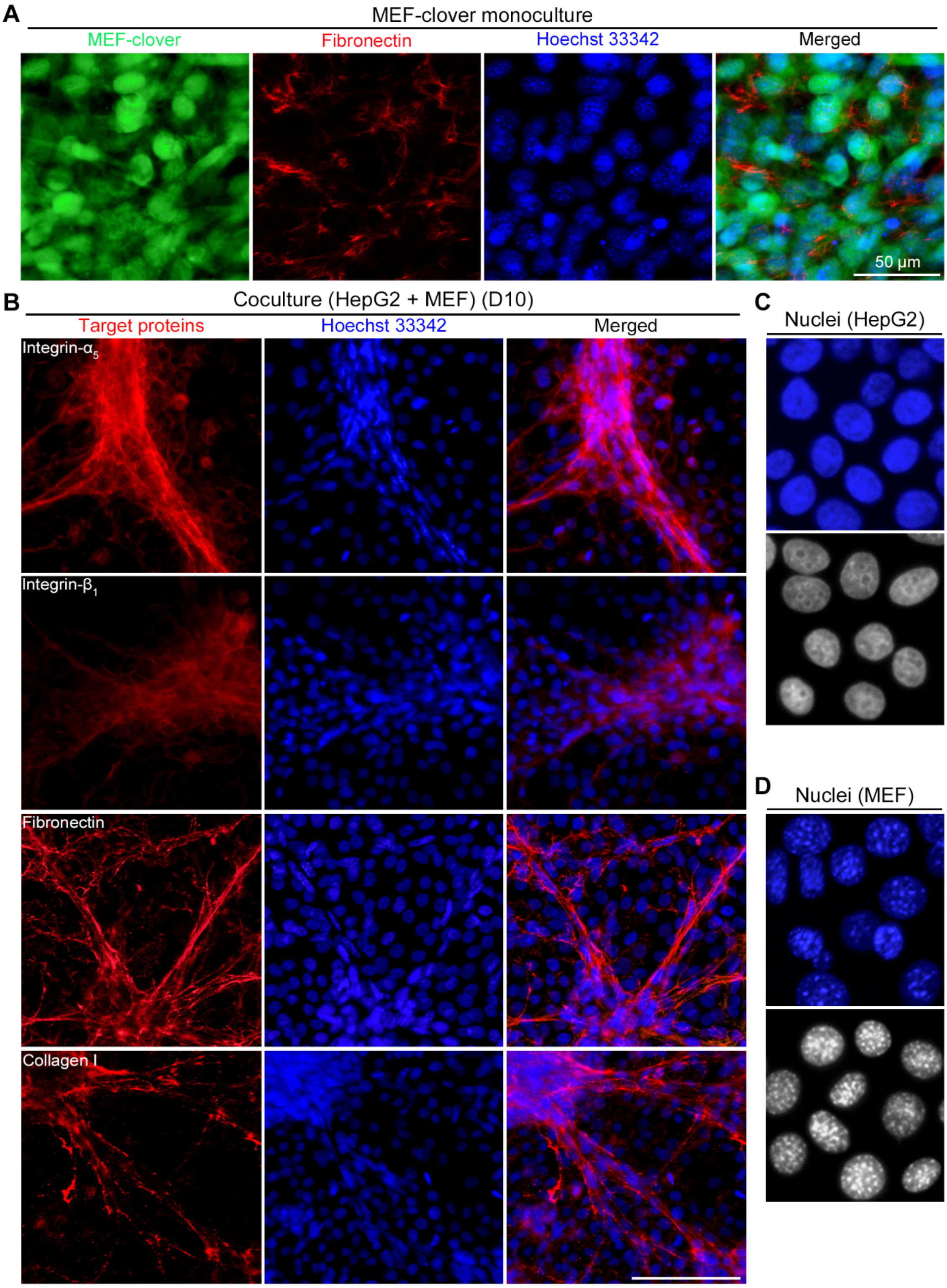

Fig. S5. No obvious fluorescence bleed-through between the green and red fluorescent channels was found. (**A**) Representative IF-staining images showing fibronectin in monocultured MEF-clover cells on day 7. Scale bar, 50 μm. (**B**) Representative IF-staining images in HepG2 cells and MEFs coculture on day 10. Scale bar, 100 μm. (**C** and **D**) Representative images showing the differences between HepG2 nuclei (**C**) and MEF nuclei (**D**) visualized using Hoechst-33342 staining.

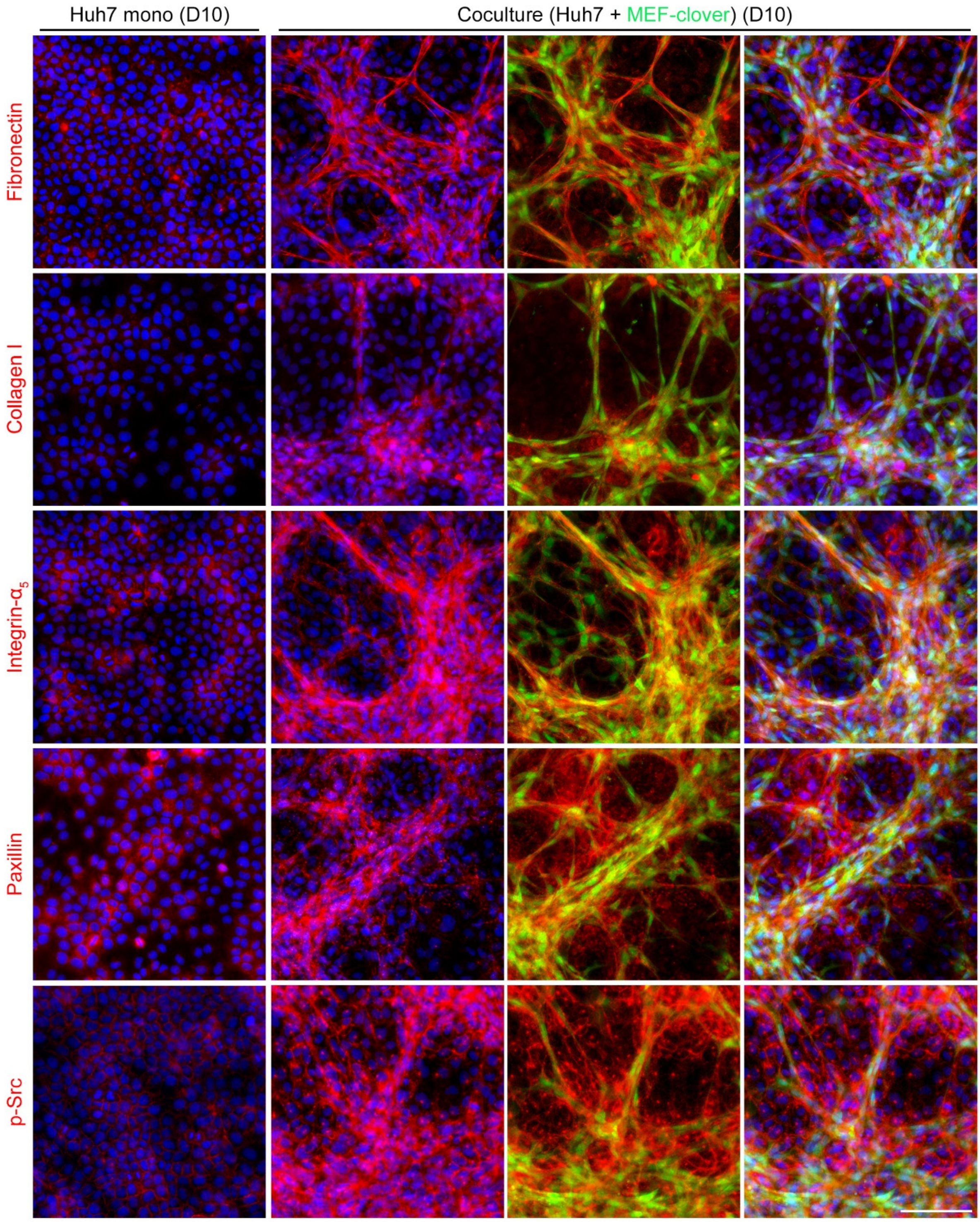

Fig. S6. ECM proteins, integrins, and their downstream molecules were enriched in 3D multilayer microstructures. Representative IF-staining images of related proteins in monocultured Huh7 cells and cocultured cells (Huh7 and MEF-clover cells) on day 10. Nuclei were visualized using Hoechst-33342. Scale bar, 100 μm.

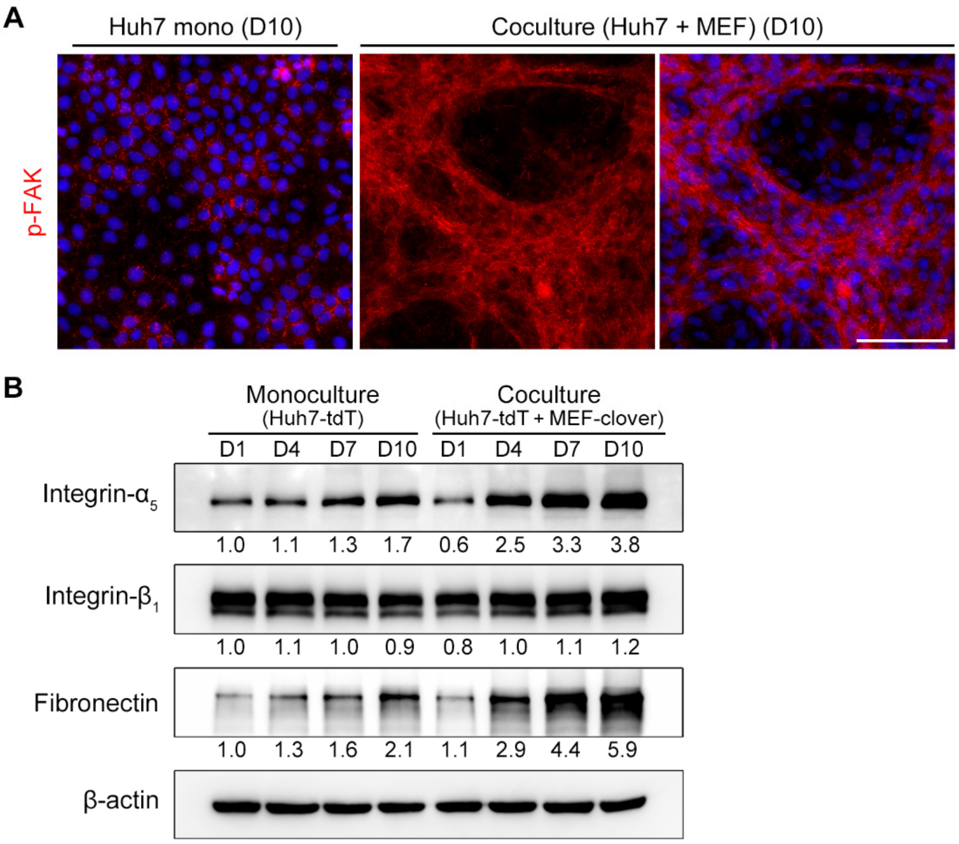

Fig. S7. Huh7 cells displayed a certain amount of fibronectin expression in monoculture. (**A**) Representative IF-staining images showing p-FAK expression in monocultured Huh7 cells and cocultured cells (Huh7 and MEF cells) on day 10. Nuclei were visualized using Hoechst-33342 staining. Scale bar, 100 μm. (**B**) WB results of the levels of integrin-α_5, integrin-β_1, and fibronectin in monocultured Huh7-tdT cells and cocultured cells (Huh7-tdT and MEF-clover cells) on day 1, day4, day7, and day10.

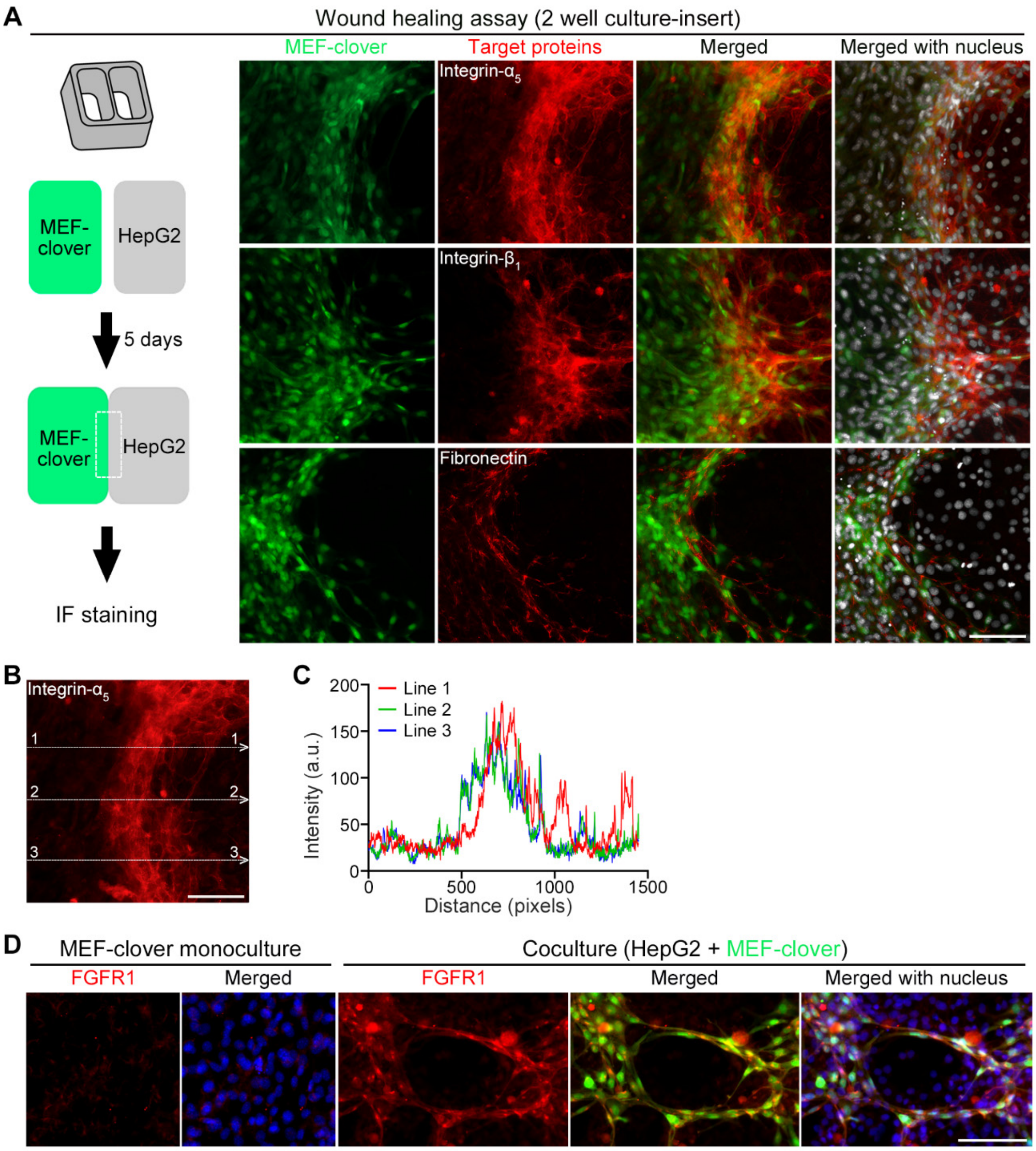

Fig. S8. Integrin clustering and fibronectin remodeling were caused by HIIs between HepG2 cells and MEFs. (**A**) Representative IF-staining images in a wound healing assay between HepG2 cells and MEF-clover cells. Two cell types were separately seeded in a 2 well culture-insert on coverslip, and the insert was removed on day 2. IF-staining was performed after the two cell types reached confluence on day 5. (**B** and **C**) Representative intensity curve showing the distribution of integrin-α_5 signal intensity after IF-staining in the above wound healing assay samples (**B**). Three intensity distribution lines were generated in an IF-staining image of integrin-α_5 by AutoQuant X3 software (**C**). (**D**) Representative IF-staining images showing FGFR1 in monocultured and cocultured MEFs. Nuclei were visualized using Hoechst-33342 staining. Scale bars, 100 μm.

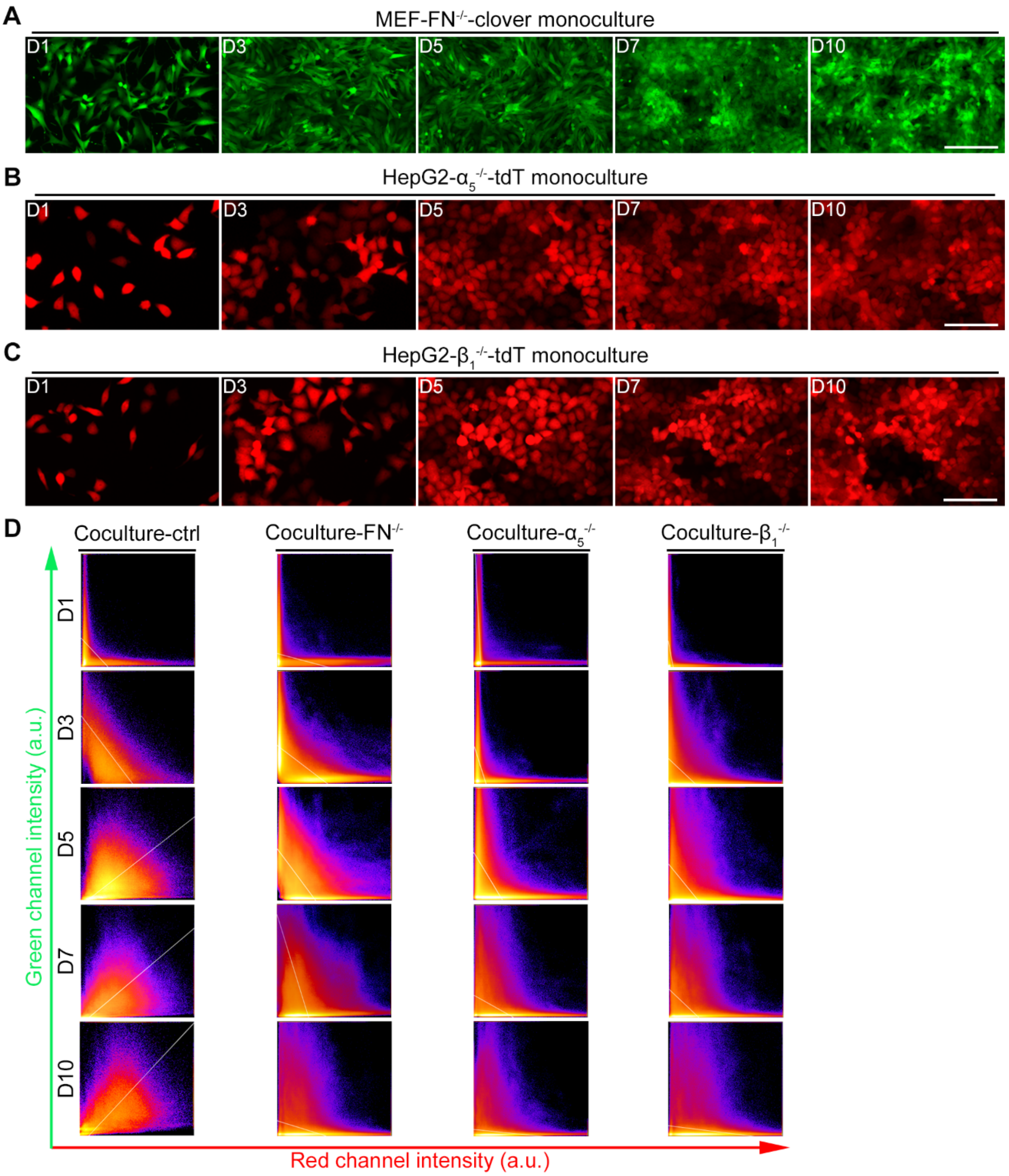

Fig. S9. 2D intensity histograms showing distributional correlation between two fluorescent cell types during different cocultures. (**A** to **C**) Representative live-cell fluorescence images of monocultured MEF-FN$^{-/-}$-clover cells (**A**), HepG2-$\alpha_5^{-/-}$-tdT cells (**B**), and HepG2-$\beta_1^{-/-}$-tdT cells (**C**) at the same spots from day 1 to day 10. (**D**) Representative 2D intensity histograms for coculture-ctrl, coculture-FN$^{-/-}$, coculture-$\alpha_5^{-/-}$, and coculture-$\beta_1^{-/-}$ groups from day 1 to day 10. The 2D intensity histograms were generated after analyzing corresponding fluorescence live-cell images with the Coloc 2 plugin of ImageJ. Scale bars, 100 μm.

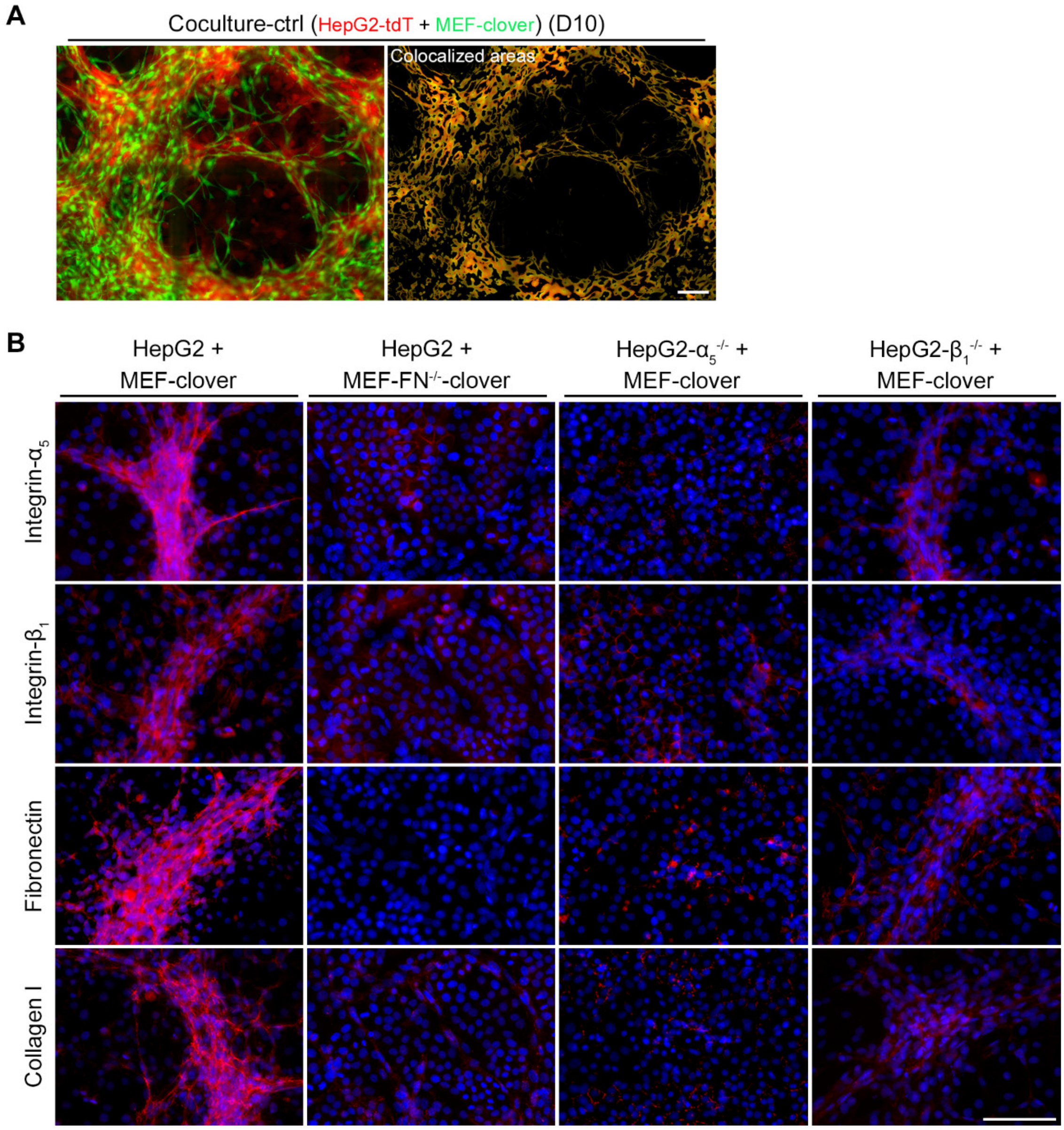

Fig. S10. Fibronectin- or integrin-α_5-knockout more effectively blocked integrin-clustering and ECM remodeling than integrin-β_1-knockout during coculture. (**A**) Representative images showing the colocalized areas in fluorescence live-cell images marked out by AutoQuant X3 software. (**B**) Representative IF-staining images on day 10. Nuclei were visualized using Hoechst-33342 staining. Scale bars, 100 μm.

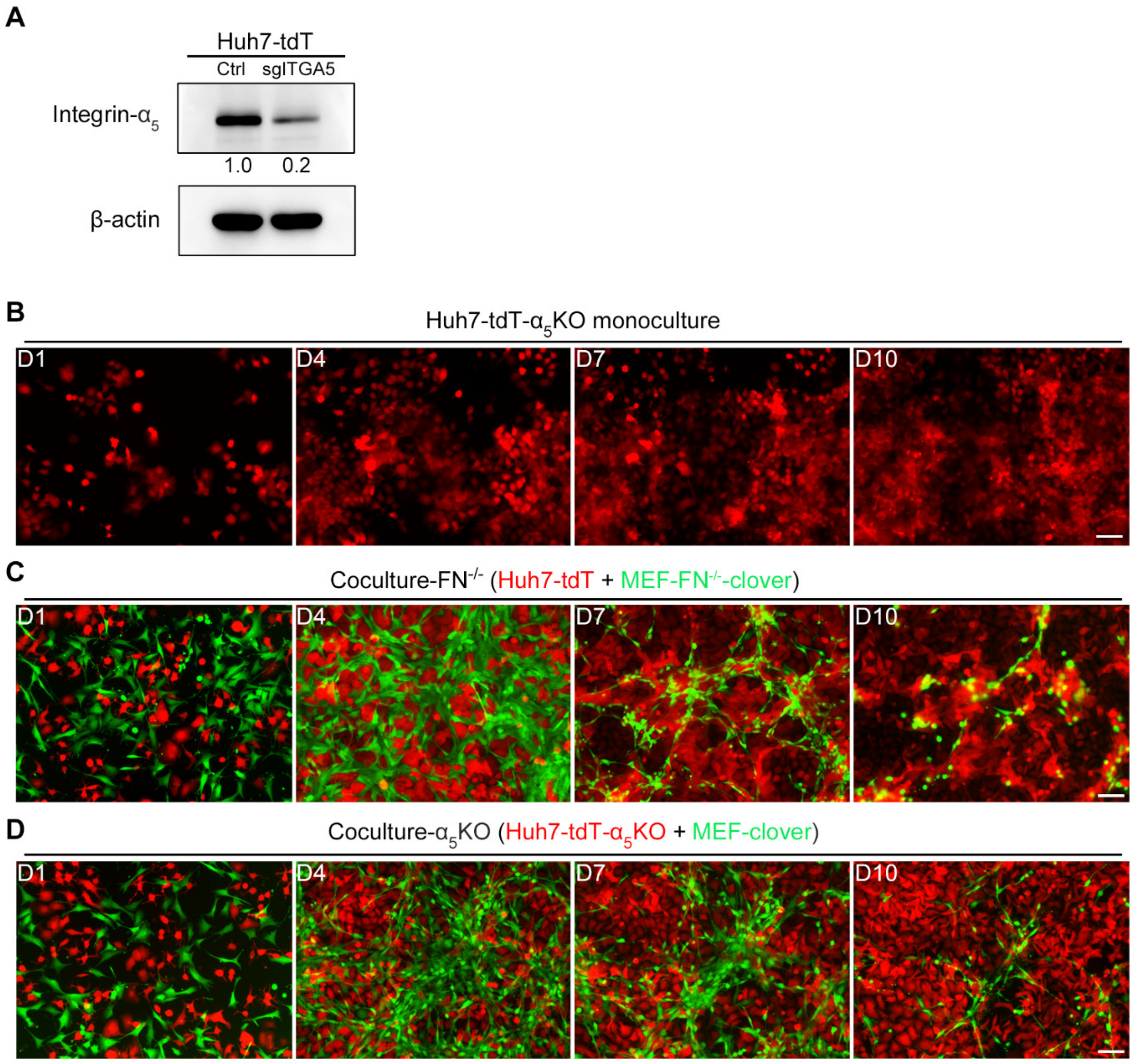

Fig. S11. Fibronectin-knockout in MEF cells or integrin-α_5-knockout in Huh7 cells interrupted the formation of 3D multilayer microstructures. (**A**) WB analysis of the levels of integrin-α_5 in Huh7-tdT cells and Huh7-tdT cells transfected with sgRNA (small guide RNA) and Cas9 targeting ITGA5 (sgITGA5). (**B**) Representative live-cell fluorescence images of monocultured Huh7-tdT-α_5KO (knockout) cells. (**C** and **D**) Representative live-cell fluorescence images of coculture-FN$^{-/-}$ (**C**) and coculture-α_5KO (**D**) groups at the same spots from day 1 to day 10. Scale bars, 100 μm.

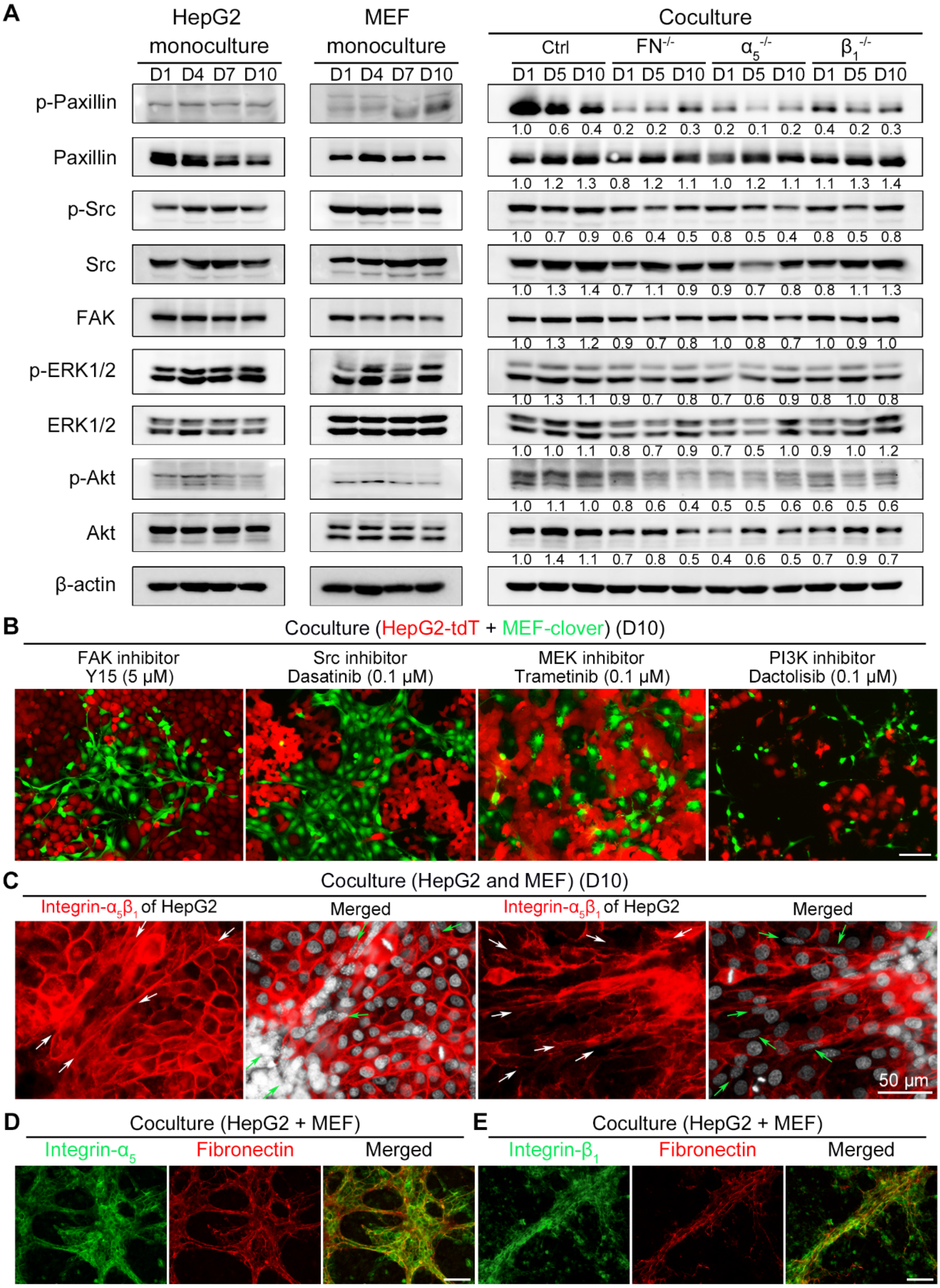

Fig. S12. Integrin downstream signaling molecule inhibitors blocked HIIs. (**A**) WB results showing the level changes of related proteins in different groups. (**B**) Representative live-cell fluorescence images showing the effects of treating coculture-ctrl group with the following inhibitors: Y15 (5 μM), dasatinib (0.1 μM), trametinib (0.1 μM), and dactolisib (0.1 μM). Inhibitors were added to the medium from day 2 to day 10, and the medium was changed daily. Scale bar, 100 μm. (**C**) Representative IF-staining images obtained using a human-specific

integrin-$\alpha_5\beta_1$ antibody (volociximab) showing the stretched morphology of HepG2 cells (white arrows) during coculture with MEFs (green arrows, based on the typical nuclei as shown in Fig. S4D) on day 10. Scale bar, 50 µm. (**D** and **E**) Representative images showing double IF-staining of the following proteins in cocultures of HepG2 cells and MEFs on day 10: integrin-α_5 and fibronectin (**D**) or integrin-β_1 and fibronectin (**E**).

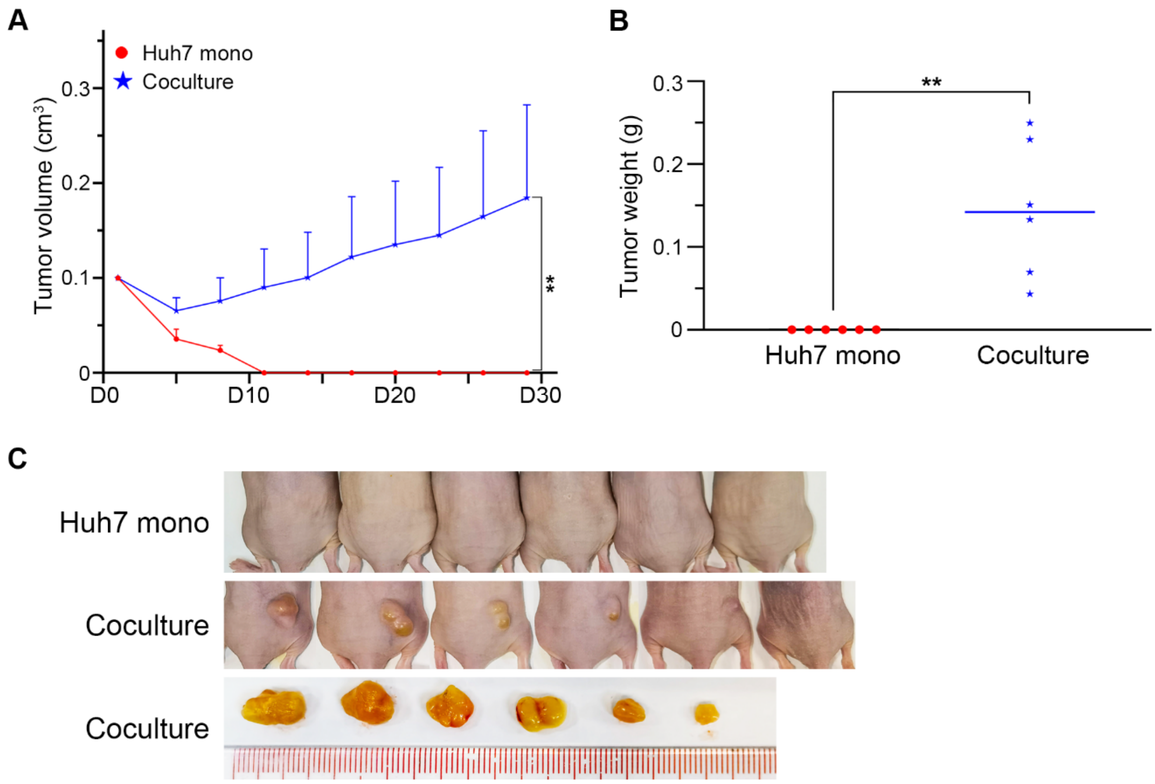

Fig. S13. HIIs-primed Huh7 cells exhibited greater tumor-formation ability than the monocultured control. Cells in each group were cultured for 4 days before subcutaneous injections. For each mouse, 2×10^6 cells in 100 µl were injected. (**A**) Quantification of the xenograft tumor volume in different groups. The tumor volume data were obtained every 3 days from day 5 to day 29 (tumor volume was assigned to 0.1 cm^3 on day 1). Unpaired t test was performed. (**B**) Quantification of the xenograft tumor weight in different groups. The tumor weight data were obtained on day 29 after sacrifice and dissection. Unpaired t test was performed. (**C**) Representative bright-field images of xenografts in different groups on day 29. All data were obtained from 6 mice in each group and are represented as the mean ± SD. ** $P<0.01$.

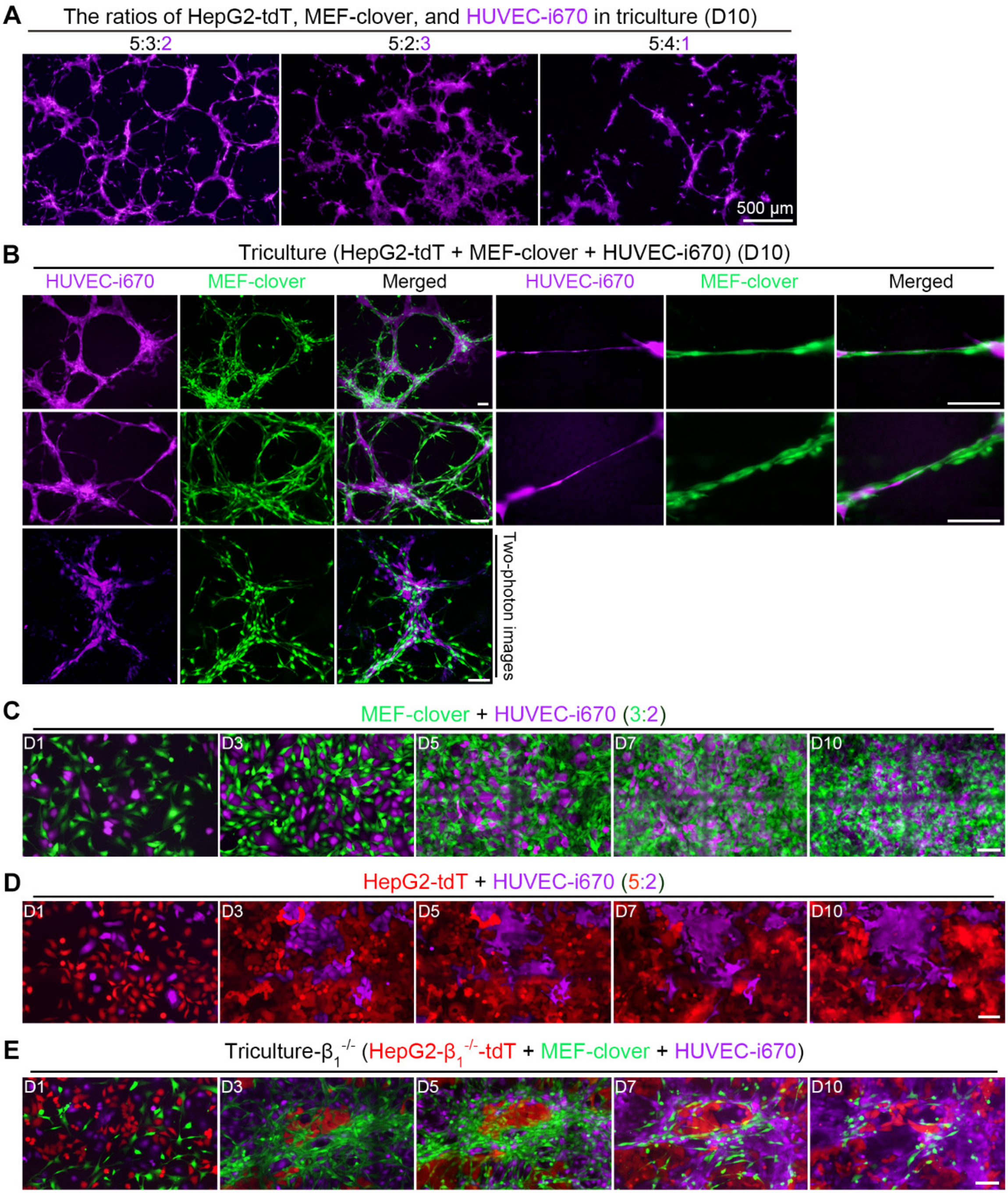

Fig. S14. The best ratio of HepG2-tdT cells, MEF-clover cells, and HUVEC-i670 cells in the triculture system was 5:3:2. (**A**) Representative live-cell fluorescence images of the far-red channel showing HUVEC-i670 cells in triculture with HepG2-tdt and MEF-clover cells at ratios of 5:3:2, 5:2:3, and 5:4:1 on day 10. Scale bar, 500 μm. (**B**) Representative live-cell fluorescence images showing the distribution of HUVEC-i670 and MEF-clover cells in triculture with HepG2-tdT cells on day 10. Scale bars, 100 μm. (**C** and **D**) Representative images of the following cocultures at the same spots: MEF-clover cells + HUVEC-i670 (**C**) or HepG2-tdT cells + HUVEC-i670 (**D**). Scale bar, 100 μm. (**E**) Representative live-cell fluorescence images of triculture-$\beta_1^{-/-}$ cells at the same spots from day 1 to day 10. Scale bar, 100 μm.

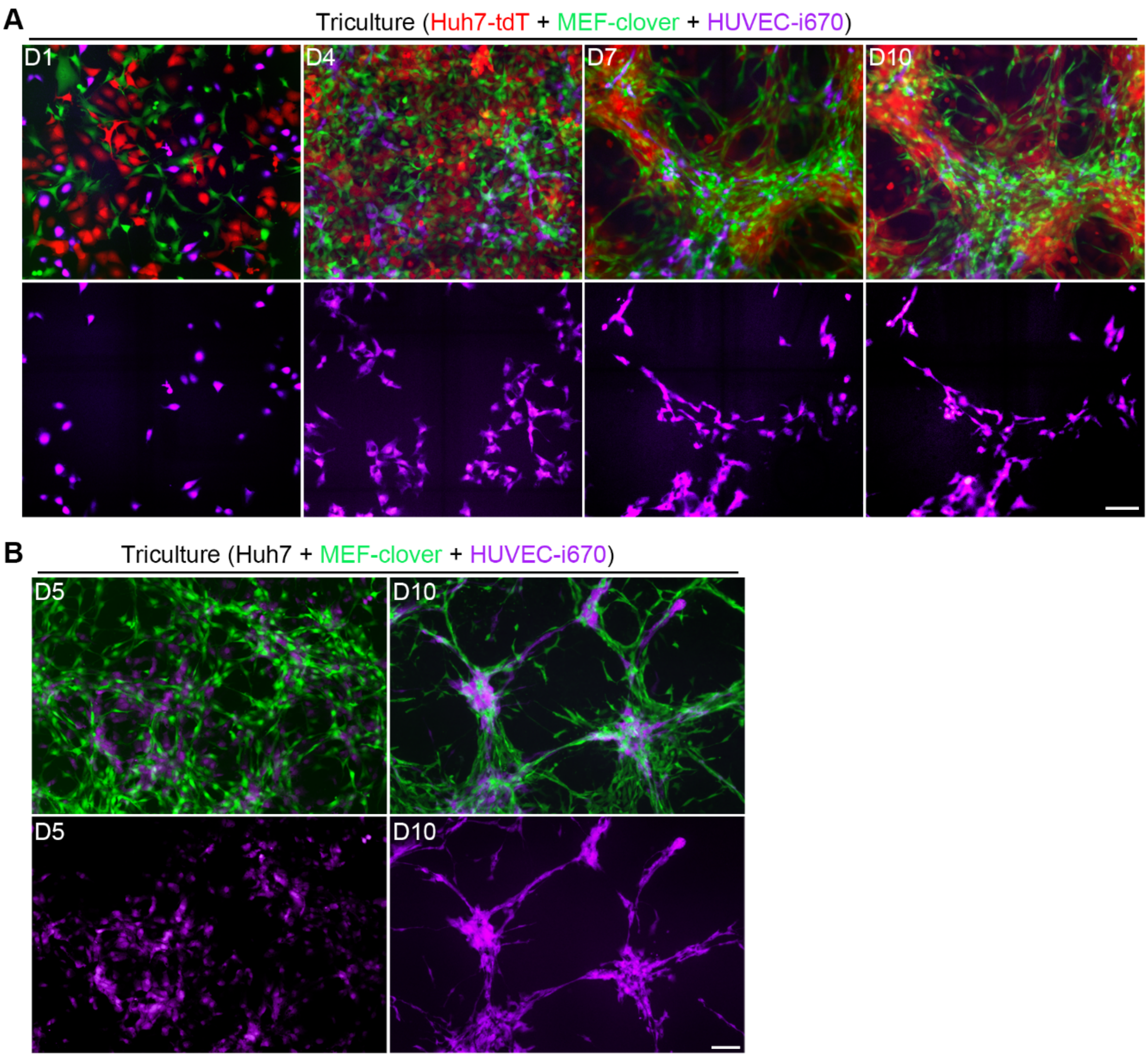

Fig. S15. HUVECs aligned to and elongated along microstructures in triculture. (**A**) Representative images showing tricultured cells at the same spots from day 1 to day 10. Corresponding HUVEC-i670 morphology is listed below. (**B**) Representative live-cell fluorescence images showing the distribution of HUVEC-i670 and MEF-clover cells in triculture with Huh7 cells on day 10. Corresponding HUVEC-i670 morphology is listed below. Scale bars, 100 µm.

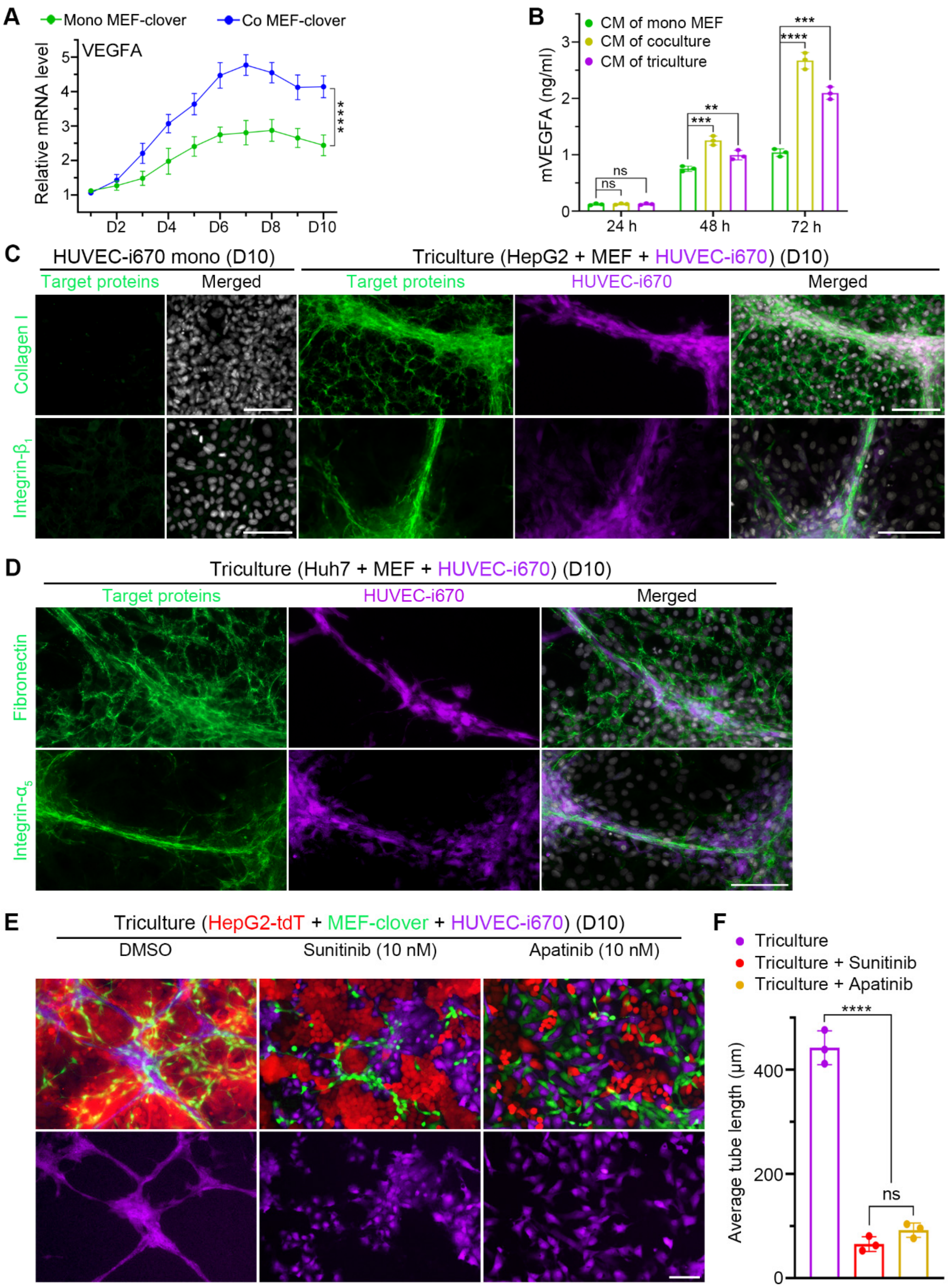

Fig. S16. The secretion of mVEGFA from MEFs was upregulated in coculture and triculture. (**A**) Relative VEGFA mRNA levels in monocultured and cocultured MEF-clover cells were analyzed using species-specific primers. *P* values were estimated using two-way ANOVA. (**B**) Mouse VEGFA (mVEGFA) levels in conditioned medium (CM) from monocultured MEF-clover, normal coculture ctrl and triculture were measured by mouse-specific ELISAs. Cells were seeded in triplicate, and CM was harvested every 24 hr. The medium was not changed to

measure cumulative mVEGFA levels. The concentration was normalized to the same number of MEF-clover cells. *P* values were estimated using two-way ANOVA. (**C** and **D**) Representative IF-staining images. (**E** and **F**) Representative images showing normal tricultured cells, and tricultured cells administered with sunitinib or apatinib. The corresponding HUVEC morphology in each group are shown below (**E**), and a plot showing the average tube length of HUVECs in these groups is listed on the right (**F**). Scale bars, 100 μm. Data are the mean ± SD from 3 independent experiments. *P* values were estimated using one-way ANOVA. * $P<0.05$, ** $P<0.01$, *** $P<0.001$, **** $P<0.0001$ and ns, not significant.

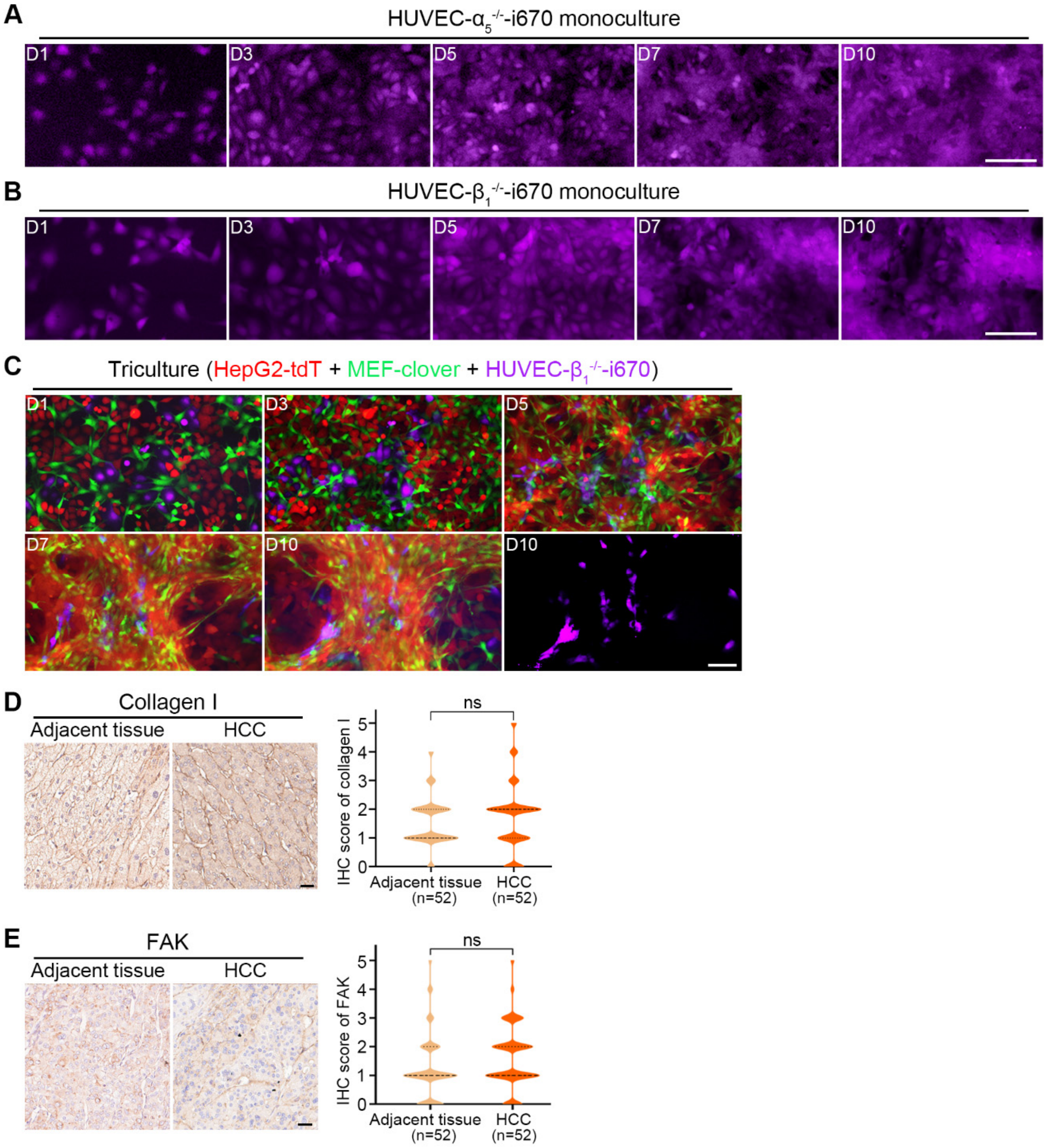

Fig. S17. Knockout of integrin-β_1 in HUVEC-i670 cells interrupted their alignment and elongation during triculture. (**A** and **B**) Representative live-cell fluorescence images of monocultured HUVECs-$\alpha_5^{-/-}$-i670 (**A**) and HUVECs-$\beta_1^{-/-}$-i670 (**B**) at the same spots from day 1 to day 10. Scale bars, 100 µm. (**C**) Representative live-cell fluorescence images showing tricultured HepG2-tdT cells, MEF-clover cells and HUVECs-$\beta_1^{-/-}$-i670 from day 1 to day 10. The morphology of HUVECs-$\beta_1^{-/-}$-i670 in triculture on day 10 is shown in the right-lower corner of this panel. Photographs were taken at the same spots. Scale bar, 100 µm. (**D** and **E**) Representative IHC-staining images of collagen I (**D**) and FAK (**E**) expression in adjacent tissues and HCC tumors from HCC patients (left) and violin plots of IHC scores from these 52 patients (right). Scale bar, 20 µm. Data are medians and quartiles. Unpaired t test was performed. ns, not significant. HR, hazard ratio.

Table S1. Main characteristics of the 52 HCC patients whose samples were used in this study.

Gender	Male	46
	Female	6
Age	≤40	8
	41-60	34
	>60	10
Cirrhosis	0	6
	1	46
Tumor size	≤5 cm	26
	>5 cm	26
Tumor differentiation	I	0
	II	9
	III	43
TNM stage	I	29
	II	5
	III	18
Microvascular invasion	0	33
	1	19

Table S2. List of antibodies.

Antibody and host	Company	Catalog #	Application and concentration
Integrin-β_1, Rabbit	CST*	34971	WB (1:2000)
Integrin-β_1, Rabbit	Abcam	ab30394	IF (1:100), IHC (1:100)
Integrin-β_1, Rat	DSHB*	AIIB2	IF (1:50)
Integrin-α_5, Rabbit	Abcam	ab150361	IF (1:200), WB (1:2000), IHC (1:100)
Integrin-α_5, Mouse	Abcam	Ab6131	IF (1:100), WB (1:1000)
Integrin-$\alpha_5\beta_1$ (Volociximab), Rabbit	Novus Biologicals	NBP2-52680	IF (1:100)
Integrin-α_v, Rabbit	Abcam	ab179475	WB (1:2000)
Fibronectin, Rabbit	Abcam	ab2413	IF (1:200), WB (1:2000), IHC (1:200)
Collagen I, Rabbit	Abcam	ab34710	IF (1:100), IHC (1:150)
Collagen I, Rabbit	CST	72026	WB (1:1000)
Paxillin, Rabbit	Abcam	ab32084	IF (1:100), WB (1:2000), IHC (1:100)
p-Paxillin (Y31), Rabbit	Abcam	ab4832	WB (1:1000)
Src, Rabbit	CST	2109	WB (1:2000), IHC (1:100)
p-Src (Y529), Rabbit	Abcam	ab32078	IF (1:100), WB (1:1000)
FAK, Rabbit	CST	3285	WB (1:1000), IHC (1:100)
FAK, Rabbit	CST	13009	WB (1:1000)
p-FAK, Rabbit	Abcam	ab81298	WB (1:1000)
Actin, Rabbit	CST	4967	WB (1:2000)
AKT, Rabbit	CST	9272	IF (1:100), WB (1:1000)
p-AKT (Ser473), Rabbit	CST	4060	WB (1:1000)
ERK1/2, Rabbit	CST	4695	WB (1: 2000)
p-ERK1/2 (Thr202/Tyr204), Rabbit	CST	4370	WB (1: 2000)
α-SMA, Mouse	CST	48938	IF (1:100), WB (1:2000)
p-VEGFR2, Rabbit	CST	2478	IF (1:100), WB (1:1000)
FGFR1, Rabbit	CST	9740	IF (1:100), WB (1:1000)
Goat anti-Rabbit IgG (H+L) 2nd Ab, Alexa-Fluor Plus 594	Invitrogen	A32740	IF (1:100)
Goat anti-Rabbit IgG (H+L) 2nd Ab, Alexa-Fluor Plus 488	Invitrogen	A32731	IF (1:100)
Goat anti-Mouse IgG (H+L) 2nd Ab, Alexa-Fluor Plus 594	Invitrogen	A11005	IF (1:100)
Goat anti-Rabbit IgG (H+L)- 2nd Ab, HRP	Bio–Rad	1706515	WB (1:5000)
Goat anti-Mouse IgG (H+L) 2nd Ab, HRP	Invitrogen	31430	WB (1:5000)

*CST, Cell Signaling Technology, Beverly, MA, USA

*DSHB, Developmental Studies Hybridoma Bank, Iowa City, IA, USA

Table S3. List of primers.

Gene name	Direction	Primer sequence (5' to 3')
ITGA5 (homo)	Forward	CAGCTCCAAGGGGAATCAGAACTC
	Reverse	TAAGTCCTGGGTGTCTGGTGCCAA
ITGA5 (mus)	Forward	CCAGATCCATGGAAGTCAGAAAGA
	Reverse	ATGCCACCTTGGGTCTGCTA
ITGB1 (homo)	Forward	TAGCGATTGAAAGGGCAATAGTT
	Reverse	GCTAGCAGGACATTTACTTTGGA
ITGB1 (mus)	Forward	GGCCAGGGCTGGTTATACA
	Reverse	CCAGCAGGCTAAACAAAGAACA
FN1 (homo)	Forward	GCTGCACATTGCCTGTTCTG
	Reverse	TCCTACAGTATTGCGGGCCA
FN1 (mus)	Forward	TCCCAGGGGAGACGTAGACT
	Reverse	TTGTGCCTCCTCTGGTTCTGCA
COL1A1 (homo)	Forward	TGGCTCTTGCAACATCTCCC
	Reverse	TCCTGACTCTCCTCCGAACC
COL1A1 (mus)	Forward	ATGGCCTCTGCAACAAACCC
	Reverse	CTTTGATACCAAACTGGGCGT
VEGFA (homo)	Forward	AGGGAAAGGGGCAAAAACGA
	Reverse	GAGGCTCCAGGGCATTAGAC
VEGFA (mus)	Forward	GAGAGGCCGAAGTCCTTTG
	Reverse	GCCATTACCAGGCCTCTTCTT
GAPDH (homo)	Forward	CCCCACCACACTGAATCTCC
	Reverse	TTGAGCACAGGGTACTTTATTGA
GAPDH (mus)	Forward	CATTTGCAGTGGCAAAGTGGAG
	Reverse	TGCATTGCTGACAATCTTGAGTGA
GAPDH (common)	Forward	GAAACTGTGGCGTGATGGC
	Reverse	GTTCAGCTCAGGGATGACCTT

Table S4. List of lentiviral plasmids.

Plasmid name	Company/Source	Vector ID
pLNT-SFFV-Clover	Addgene	#87216
LeGO-T2	Addgene	#27342
pLV[Exp]-Puro-EF1A>[iRFP670]	Vectorbuilder	VB180918-1138aba
pLV[CRISPR]-hCas9:T2A:Puro-U6 >hITGB5[gRNA#4971]	Vectorbuilder	VB191030-3339urz
pLV[CRISPR]-hCas9:T2A:Puro-U6 >hITGB1[gRNA#3099]	Vectorbuilder	VB191107-1132gee
